# Milk-derived exosomes as functional nanocarriers in wound healing: Mechanisms, applications, and future directions

**DOI:** 10.1016/j.mtbio.2025.101715

**Published:** 2025-03-28

**Authors:** Jing Ruan, Yuping Xia, Yilei Ma, Xiyao Xu, Shihao Luo, Jia Yi, Baihui Wu, Rongbing Chen, Hanbing Wang, Honggang Yu, Qinsi Yang, Wei Wu, Da Sun, Junbo Zhong

**Affiliations:** a Institute of Life Sciences & Biomedical Collaborative Innovation Center of Zhejiang Province, Wenzhou University, Wenzhou 325035, China; b Department of Burn and Plastic Surgery, Zigong Fourth People's Hospital, Zigong 643099, China; c Department of Biomedical Engineering, City University of Hong Kong, 999077, Hong Kong Special Administrative Region of China; d Department of Biotechnology, The University of Hong Kong, 999077, Hong Kong Special Administrative Region of China; e Hand and Foot Surgery, The Affiliated Yiwu Hospital of Wenzhou Medical University, Yiwu 322000, China; f Wenzhou Institute, University of Chinese Academy of Sciences, Wenzhou 325001, China; g Key Laboratory for Biorheological Science and Technology of Ministry of Education, Bioengineering College of Chongqing University, Chongqing 400044, China; h Jin Feng Laboratory, Chongqing, 401329, China

**Keywords:** Milk-derived exosomes, Wound healing, Exosome isolation, Cell-free therapy, Inflammation regulation

## Abstract

Wound healing presents a significant challenge in healthcare, imposing substantial physiological and economic burdens. While traditional treatments and stem cell therapies have shown benefits, milk-derived exosomes (MDEs) offer distinct advantages as a cell-free therapeutic approach. MDEs, isolated from mammalian milk, are characterized by their biocompatibility, ease of acquisition, and high yield, making them a promising tool for enhancing wound repair. This review provides a comprehensive analysis of the composition, sources, and extraction methods of MDEs, with a focus on their therapeutic role in both acute and diabetic chronic wounds. MDEs facilitate wound healing through the delivery of bioactive molecules, modulating key processes such as inflammation, angiogenesis, and collagen synthesis. Their ability to regulate complex wound-healing pathways underscores their potential for widespread clinical application. This review highlights the importance of MDEs in advancing wound management and proposes strategies to optimize their use in regenerative medicine.

## Highlights

• MDEs as advanced nanocarriers for cell-free therapies.
• MDEs boost wound healing via angiogenesis and collagen synthesis.
• MDEs' targeted bioactive molecule delivery speeds up diabetic wound repair.
• MDEs enhance platelet activation and hemostasis in acute wound care.
• MDE-based nanomedicines offer regenerative potential across disciplines.

## Graphical abstract

**Figure:**
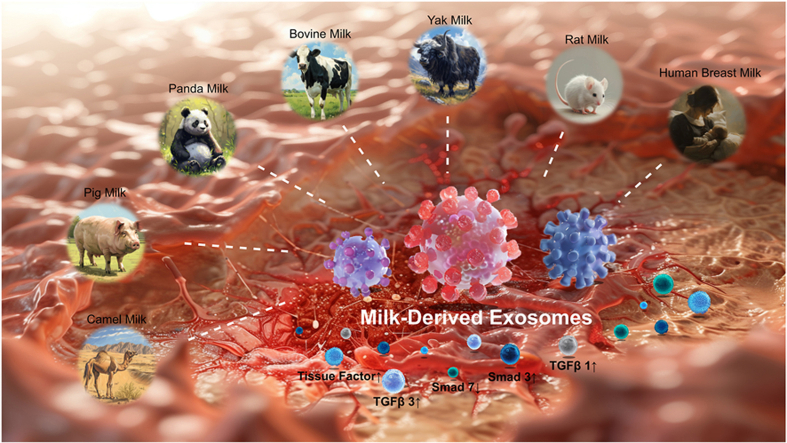

## 1 Introduction

Wound healing remains a major challenge in global healthcare, particularly with the increasing prevalence of chronic wounds [1]. In 2021, there were 529 million people with diabetes worldwide, with a global age-standardized overall prevalence of diabetes of 6.1%, and it is projected that more than 1.31 billion people will have diabetes by 2050 [2]. The skin, as the body's largest organ, plays a critical role in maintaining homeostasis, acting as a barrier against pathogens, and regulating temperature [3]. Disruption of skin integrity, whether due to trauma, disease, or diabetes mellitus (DM), can result in delayed healing, leading to complications such as infection, prolonged inflammation, and scarring [4]. Among these, diabetic foot ulcers affect over 30% of DM patients, with a high recurrence rate (40% within one year, 65% within five years) [5]. Due to the impaired healing mechanisms and the complexity of the wound microenvironment in diabetic patients, successful tissue regeneration is extremely difficult to achieve [6]. These wounds often develop into chronic, non-healing ulcers, presenting complications such as infection, amputation, and even mortality [7].

Traditional therapies, including the use of antibiotics and topical treatments, are often ineffective in treating chronic wounds due to issues such as frequent dressing changes, infection risks, and prolonged healing times [8,9]. Moreover, stem cell-based treatments, which have been explored for their potential to promote healing through growth factors and cytokines, are limited by concerns over immune rejection, infection risks, and ethical challenges [10,11]. Therefore, there is a growing need for advanced, cell-free therapeutic options. In contrast, milk-derived exosomes (MDEs) offer a promising alternative with distinct advantages in efficacy, safety, and cost-effectiveness. Exosomes, nanosized vesicles (30–150 nm), are secreted by various cell types and facilitate intercellular communication [[12], [13], [14]]. These vesicles carry bioactive molecules such as microRNAs (miRNAs), proteins, and lipids, which are crucial for processes like angiogenesis, extracellular matrix (ECM) remodeling, and immune regulation, all key aspects of wound healing [15]. The ability of exosomes to modulate these pathways positions them as a non-cellular therapeutic alternative with minimal risk of immune rejection [[16], [17], [18]].

Compared to other exosome sources, such as plant-derived and blood-derived exosomes, MDEs have attracted significant attention due to their scalability, safety, and biocompatibility [19]. Plant-derived exosomes, while cost-effective and abundant, often face challenges related to their stability and immunogenicity in mammalian systems, while blood-derived exosomes have shown potential in wound healing due to their ability to carry growth factors and cytokines [20]. However, their isolation is often complicated by the presence of contaminants like lipoproteins and other extracellular vesicles (EVs), which can affect their purity and therapeutic efficacy. Mammalian milk, particularly bovine and human milk, provides an abundant source of exosomes, which contain various bioactive molecules that facilitate cellular functions such as proliferation, migration, and angiogenesis, making them highly suitable for therapeutic applications [21,22]. Compared to traditional therapies, MDEs demonstrate superior efficacy in preclinical models, with studies showing accelerated wound closure rates, enhanced angiogenesis, and reduced inflammation [23]. Furthermore, the production of MDEs is cost-effective, as milk is an abundant and inexpensive resource, making them more accessible than stem cell-based therapies, which require complex and expensive manufacturing processes [24]. Compared with other cell-free therapies, MDEs are naturally derived, exhibit high biocompatibility and low immunogenicity, minimize the risk of adverse immune reactions, and have high intrinsic stability, allowing application in challenging physiological environments [25]. Moreover, their ability to efficiently deliver therapeutic agents such as miRNAs and siRNAs has shown promise in preclinical models, particularly in reducing oxidative stress and promoting angiogenesis in diabetic wounds [26]. These advantages position MDEs as a viable and innovative therapeutic option for wound management (Table 1). However, it is important to note that, to date, no human clinical trials have been conducted to explore the efficacy of MDEs in wound healing, indicating a crucial area for future research.

**Table 1:** Comparison of MDEs with traditional therapies and stem cell-based treatments.

Aspect	Traditional Therapies	Stem Cell-Based Treatments	MDEs
Efficacy	Limited efficacy in chronic wounds, prolonged healing times and high recurrence rates.	High potential for tissue regeneration but variable efficacy due to immune rejection risks.	Demonstrated efficacy in promoting angiogenesis, reducing inflammation, and enhancing wound healing.
Safety	Risk of infection, antibiotic resistance, and frequent dressing changes.	Risk of immune rejection, tumorigenicity, and infection from cell-based approaches.	Low immunogenicity, minimal risk of adverse immune reactions, and high biocompatibility.
Cost-Effectiveness	Moderate cost but high long-term expenses due to prolonged treatment and complications.	High cost due to complex production, storage, and ethical/regulatory challenges.	Cost-effective due to scalable production from abundant milk sources and minimal processing needs.
Scalability	Limited by the need for frequent clinical interventions.	Limited by ethical concerns, donor availability, and complex manufacturing processes.	Highly scalable due to the abundance of milk as a natural source and straightforward isolation methods.
Regulatory Hurdles	Well-established but limited by antibiotic resistance and side effects.	Significant regulatory challenges due to ethical concerns and safety risks.	Fewer regulatory hurdles due to non-cellular nature and low immunogenicity.

**Fig. 1:**
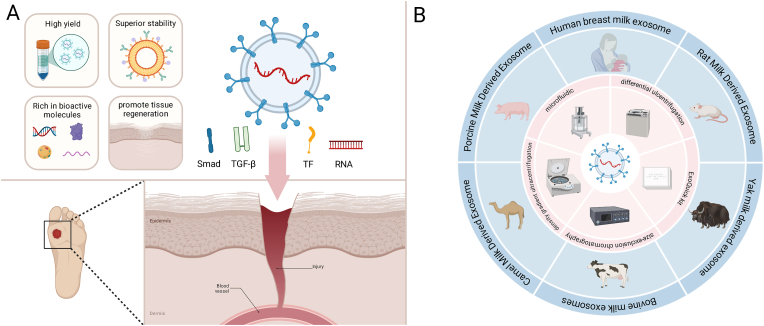
Schematic diagram. A) Milk-derived exosomes (MDEs) have unique biological advantages and can promote wound healing through a variety of bioactive components. B) Rich sources and various isolation methods of MDEs.

This review aims to explore the potential of MDEs in wound healing, with a focus on their molecular composition, mechanisms of action, and their role in treating both acute and chronic wounds, particularly in diabetic populations (Fig. 1A). We will also discuss current methodologies for the isolation and characterization of MDEs (Fig. 1B), and evaluate their future prospects in regenerative medicine.

## 2 Composition of MDE

**Fig. 2:**
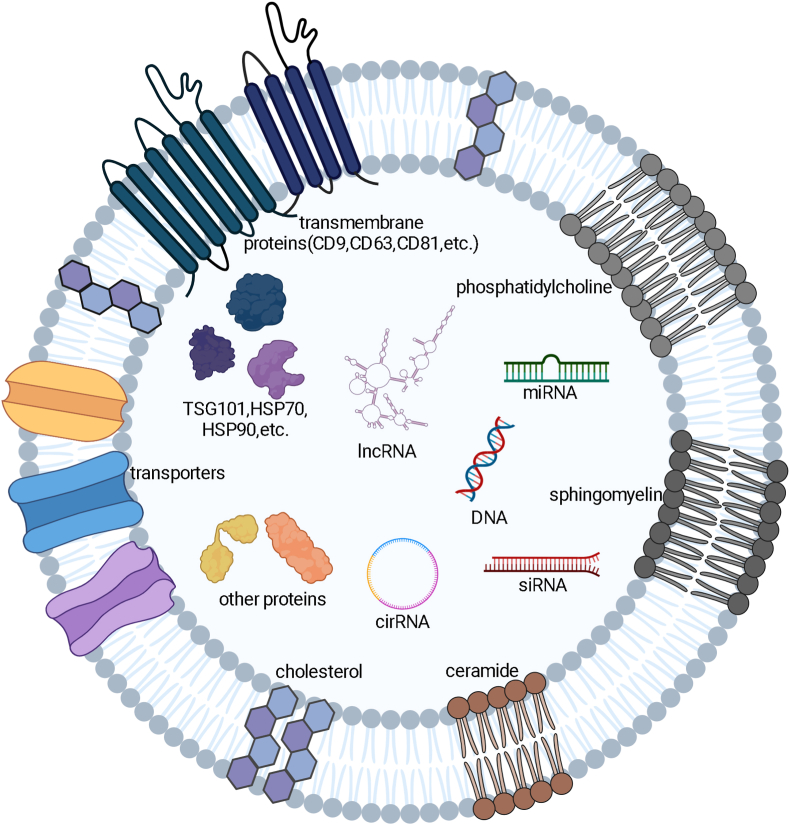
MDEs are composed of a variety of lipids, proteins and nucleic acids, which play a role in biological activity.

Mammalian milk is a highly complex heterogeneous fluid, containing a diverse array of bioactive components vital for numerous physiological functions, including the development of the immune and nervous systems, as well as metabolic processes [27]. Within this milieu, MDEs have emerged as a significant therapeutic vehicle due to their composition, which includes lipids, proteins, and nucleic acids (Fig. 2), all of which contribute to their roles in intercellular communication and regenerative medicine [28].

### 2.1 Lipids in MDE

Lipids are essential components of MDEs, contributing to both their structural integrity and their diverse biological functions [29]. The lipid composition not only stabilizes the exosomal membrane bilayer structure but also plays a critical role in exosome biogenesis, intercellular communication, and therapeutic delivery [28]. Understanding the specific lipid profile of MDEs, including cholesterol, phosphatidylcholine (PC), sphingomyelin (SM), and ceramide, is vital for optimizing their potential as therapeutic agents.

#### 2.1.1 Structure and role of lipids in exosome formation and stability

The lipid bilayer of MDEs primarily comprises phospholipids, cholesterol, and sphingolipids, which together provide the necessary membrane fluidity and stability [30]. PC and SM are abundant on the outer leaflet of the bilayer, contributing to membrane integrity [29]. PC is particularly important for structural stability, while SM regulates membrane permeability, ensuring MDEs can survive extracellular environments [31]. These lipids are integral for protecting exosomes as they travel through the body, enabling effective delivery of therapeutic cargo [32].

Cholesterol, distributed throughout the membrane, plays a critical role in maintaining the fluidity and stability of MDEs [33]. It enhances the vesicle's ability to fuse with target cell membranes, facilitating the transfer of bioactive molecules such as proteins and nucleic acids [34]. The cholesterol content in exosomes has been linked to their capacity for membrane fusion, making it a key factor in the successful delivery of encapsulated molecules in therapeutic contexts [35].

Moreover, the size and morphology of MDEs, typically ranging from 30 to 150 nm in diameter and spherical in shape, further enhance their therapeutic effectiveness. The nanoscale size allows for deep tissue penetration and efficient uptake by target cells within the wound site, essential for the delivery of their bioactive cargo [36]. However, maintaining uniform size distribution and structural integrity is vital. Aggregation or structural alterations can impede MDE stability and functional delivery, ultimately affecting their capacity to promote healing. Thus, the synergy between the lipid composition and physical characteristics of MDEs underpins their role in effective wound healing therapies.

#### 2.1.2 Ceramide: A critical Lipid in exosome biogenesis

One of the most functionally significant lipids in MDEs is ceramide, which is central to exosome biogenesis [37]. Ceramide triggers membrane invagination and drives the formation of exosomes through the endosomal sorting complex required for transport-independent pathway [38]. This process is crucial for the encapsulation of bioactive molecules into exosomes, ensuring that therapeutic agents can be effectively delivered to target cells. Additionally, ceramide influences the curvature of the membrane, allowing for the efficient packaging of molecules within MDEs [39].

Ceramide also plays a regulatory role in determining exosomal cargo, making it indispensable for the therapeutic applications of MDEs [37]. Without ceramide, the exosomal formation process would be impaired, affecting the quantity and quality of the bioactive materials these vesicles can carry.

#### 2.1.3 Phosphatidylserine (PS) and Phosphatidylethanolamine (PE): targeting and uptake

The lipid composition of MDEs, particularly the presence of PS on their surface, plays a crucial role in targeting and uptake by recipient cells at the wound site [31]. This lipid not only facilitates MDE recognition and engulfment by macrophages and other cells involved in healing but also ensures that the therapeutic cargo of miRNAs and proteins is efficiently delivered to where it is most needed, thus enhancing the wound healing process [40]. PE, another lipid enriched in MDEs, contributes to membrane fusion and exosomal flexibility, which are essential for efficient intracellular delivery [41]. Both PS and PE show enrichment in specific species and lactation stages, further underscoring the adaptability of MDEs for therapeutic delivery in different physiological environments [42].

#### 2.1.4 Functional implications of Lipid composition for therapeutic delivery

The unique lipid composition of MDEs directly influences their effectiveness as therapeutic delivery vehicles [43,44]. The high concentration of cholesterol, ceramide, and SM stabilizes MDEs in circulation, ensuring that they remain intact long enough to reach target tissues. Furthermore, the presence of PS on the surface facilitates targeted uptake by recipient cells, enhancing the therapeutic potential of MDEs in applications such as wound healing and tissue regeneration [31].

Each lipid class, including phospholipids, cholesterol, sphingolipids, and ceramide, contributes to different aspects of MDE formation, stability, and therapeutic delivery [31]. By understanding how these lipids interact and influence exosome function, researchers can further optimize MDEs for clinical applications, enhancing their effectiveness as drug delivery systems in regenerative medicine.

### 2.2 Proteins in MDE

Proteins encapsulated in MDEs are essential to their structural integrity, functional roles, and therapeutic potential. The diverse protein composition of MDEs, encompassing proteins involved in membrane fusion, exosome biogenesis, and signal transduction, contributes to their immunomodulatory, regenerative, and therapeutic capabilities, making them a valuable tool in applications such as wound healing and tissue regeneration [45,46].

#### 2.2.1 Structural proteins and exosome biogenesis

Proteins such as tetraspanins (e.g., CD9, CD63, CD81), which are highly enriched in MDEs, play a central role in maintaining their structural formation and exosome biogenesis, by acting as scaffolding proteins, facilitating membrane organization, vesicle budding, and cargo sorting. A study by Giovanazzi et al. revealed the presence of CD3, CD14, CD9, CD24, CD29, CD44, CD63, CD105, CD133-1, CD146, CD326, and CD81 in human breast MDEs (HBM-Exos) [47]. Tetraspanins CD63 and CD81, in particular, are recognized as exosomal markers and are critical in defining the exosomal identity [28]. The presence of these proteins ensures the structural stability of MDEs, which is essential for their long-term circulation and functionality in therapeutic delivery.

Additionally, proteins such as ALG-2 interacting protein X (Alix) and tumor susceptibility gene 101 (TSG101) are involved in exosomal cargo sorting and membrane trafficking [48]. These proteins help ensure that bioactive molecules, including miRNAs and enzymes, are efficiently packaged within exosomes during their formation. This selective cargo loading is a critical step in exosome-mediated communication between cells and enhances the therapeutic utility of MDEs in regenerative medicine.

#### 2.2.2 Functional proteins and signal transduction

MDEs also carry essential cytoskeletal proteins such as actin, tubulin, and cofilin, which are crucial for enhancing cell migration and adhesion, fundamental processes in wound re-epithelialization and tissue regeneration [49,50]. By transporting these proteins directly to the wound site, MDEs actively contribute to the cellular dynamics essential for effective wound healing. Heat shock proteins, found in MDEs, act as molecular chaperones, aiding in protein folding and protecting cells from stress-induced damage [51]. These proteins also play a crucial role in enhancing MDE stability and function under physiological stress conditions, making them particularly useful in clinical applications where MDEs must remain effective in challenging environments.

#### 2.2.3 Proteomic Diversity and therapeutic potential

The protein composition of MDEs can vary depending on the source (e.g., human, bovine) and the lactation stage. For instance, colostrum-derived exosomes (Col-MDEs) are rich in proteins associated with immune responses, including those involved in antimicrobial activity and complement activation. In contrast, Mature-MDEs are predominantly involved in transport and apoptosis, reflecting the dynamic nature of MDE proteomics across different developmental stages [52,53]. This variation in protein content can be leveraged to tailor MDEs for specific therapeutic interventions, such as promoting wound healing or enhancing tissue regeneration in patients with chronic conditions [31].

Interestingly, certain surface markers typically found in other EVs, such as integrin-β1, P-selectin, and ER marker calnexin, are absent in MDEs [54]. This distinction underscores the unique proteomic identity of MDEs, contributing to their low immunogenicity and high biocompatibility, which are critical for their success in therapeutic delivery applications.

The presence of key proteins such as tetraspanins, Alix, and cytoskeletal elements ensures the stability and functionality of MDEs in diverse physiological environments. Furthermore, the dynamic protein composition of MDEs, which changes across lactation stages, opens avenues for customizing exosome-based therapies to address specific clinical needs, particularly in immune modulation and tissue regeneration.

### 2.3 Nucleic acids in MDE

Nucleic acids encapsulated in MDEs play critical roles in gene regulation, immune function, and therapeutic potential. The primary nucleic acids found in MDEs include miRNAs, long non-coding RNAs (lncRNAs), circular RNAs (circRNAs) [55]. These molecules exhibit remarkable stability in harsh physiological conditions, such as low pH and RNase activity, which makes MDEs ideal vectors for therapeutic delivery [56,57]. Quantitative studies have shown that the concentration of these nucleic acids can vary based on the species, lactation stage, and environmental factors, influencing their biological and therapeutic functions [58].

#### 2.3.1 miRNAs: key regulators of immune response and wound healing

mRNAs are the most abundant class of nucleic acids in MDEs, serving as pivotal regulators composed of 19–24 nucleotides, regulating key biological processes such as immune responses, cell proliferation, and tissue repair [[59], [60], [61]]. Quantitative analysis has revealed that exosomal miRNAs can make up to 10–20% of the total RNA content in HBM-Exos. In bovine MDEs (BM-Exos), studies have identified over 3000 distinct miRNAs, many of which are involved in immune modulation and metabolic regulation [62,63]. In addition, hsa-miRNA-148a-3p was the dominant miRNA in HBM-Exos, while in another study, the miRNA types were hsa-miRNA-200c-3p, hsa-miRNA-148a-3p, hsa-let-7i-5p, hsa-miRNA-146b-5p, hsa-miRNA-200a-3p, hsa-miRNA-30a-5p, hsa-miRNA-21-5p, hsa-miRNA-26a-5p, hsa-let-7f-5p and hsa-miRNA-146a-5p showed their presence levels from high to low, respectively. These miRNAs, such as miR-15b, miR-27b, and miR-106b, are enriched in colostrum and have been linked to neonatal immune development [61].

Among the diverse miRNAs in MDEs, miR-223 plays a pivotal role in modulating the immune response and enhancing wound healing [64]. Delivered by MDEs, miR-223 suppresses excessive inflammation, promoting faster wound closure with reduced scarring [65,66]. This miRNA targets pathways that regulate pro-inflammatory cytokines, thus facilitating an optimal environment for wound repair.

#### 2.3.2 lncRNAs and circRNAs: enhancing cellular communication and tissue repair

lncRNAs and circRNAs in MDEs serve as critical regulators of gene expression and cellular communication [67]. lncRNAs, typically longer than 200 nucleotides, have been identified in significant quantities across various mammalian species [55]. In BM-Exos, over 3475 novel lncRNAs have been identified, many of which are involved in immune function, osteoblast differentiation, and neurodevelopment [55]. The expression levels of these lncRNAs vary across lactation stages, with colostrum exhibiting higher levels of immune-related lncRNAs, highlighting their potential for enhancing immune defense and tissue regeneration [68].

circRNAs, with their stable, closed-loop structures, are resistant to exonuclease degradation, making them particularly valuable for long-term gene regulation [69]. Over 2059 circRNAs have been identified in BM-Exos, many of which regulate immune pathways and are involved in tissue repair processes [70]. These circRNAs act as “sponges” for miRNAs, modulating their activity and enhancing the therapeutic efficiency of MDEs in regenerative medicine [71].

#### 2.3.3 Quantitative insights into therapeutic applications

The unique nucleic acid profile of MDEs, particularly their high content of miRNAs, lncRNAs, and circRNAs, makes them highly suitable for therapeutic applications. Quantitative studies in animal models have shown that the oral administration of BM-Exos leads to a significant increase in circulating miRNAs, with up to 13 immune-related miRNAs showing a 2–3 fold increase in serum levels after ingestion [72,73]. This highlights the potential for oral delivery of therapeutic MDEs to modulate immune responses and promote tissue regeneration in clinical settings.

Additionally, the selective packaging of nucleic acids within MDEs, such as the enrichment of immune-related miRNAs in colostrum-ensures targeted therapeutic effects [45]. For instance, miR-148a-3p has been shown to target IKBKB, thereby suppressing NF-κB signaling and its associated inflammatory responses, while miR-30b-5p plays a role in promoting cellular invasion and immunosuppression by targeting GalNAc-transferases [20]. These data underscore the therapeutic versatility of MDEs, particularly in immune modulation, tissue repair, and regenerative medicine.

## 3 Origin of MDE

### 3.1 MDE from different species

**Fig. 3:**
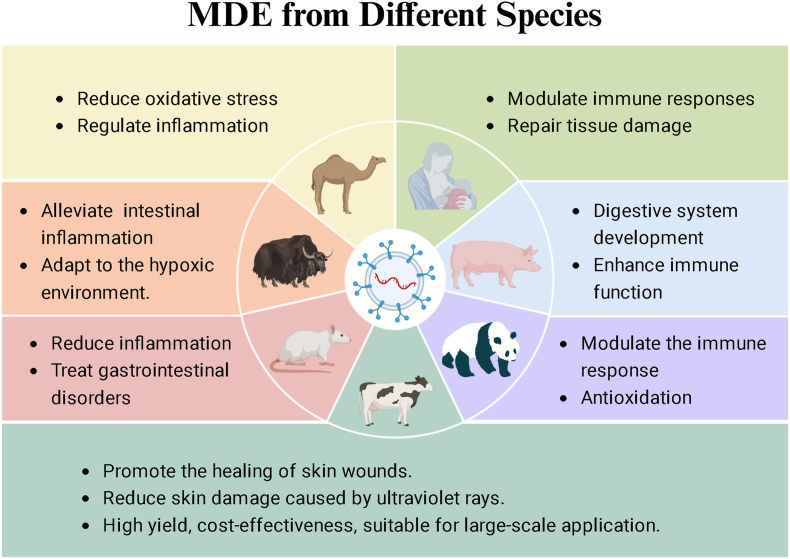
MDEs from various species, including bovine, human, camel, yak, pig, panda and rat, have shown unique advantages and broad therapeutic prospects.

MDEs from different species exhibit significant potential in promoting wound healing and tissue regeneration, owing to their diverse bioactive cargo. Key exosomes from bovine, human, camel, yak, and panda milk have shown therapeutic promise in various models [19]. Among them, BM-Exos are the most widely studied, which are favored for their high yield, cost-effectiveness, and suitable for large-scale application. HBM-Exos have powerful immunomodulatory properties, but are limited by their availability. Camel MDEs (CM-Exos) are particularly effective in alleviating oxidative stress and inflammation, while yak MDEs (YM-Exos) show promise under hypoxic conditions. Porcine MDEs (PM-Exos) and panda MDEs (PAM-Exos) have been less studied, but may have unique therapeutic benefits under specific circumstances (Fig. 3).

#### 3.1.1 BM-Exos

BM-Exos are the most extensively studied due to their abundance and ease of extraction. BM-Exos promote fibroblast proliferation by approximately 30–40% and increase collagen deposition by over 50%, significantly accelerating wound closure in full-thickness wound models [74,75]. Additionally, BM-Exos reduce reactive oxygen species (ROS) production in skin cells exposed to ultraviolet (UV) damage, helping to protect keratinocytes from oxidative stress [76].

#### 3.1.2 HBM-Exos

HBM-Exos have demonstrated strong potential in immune regulation and wound healing [77]. Enriched with immune-related miRNAs, such as miR-223, HBM-Exos reduce inflammatory markers, significantly improving wound healing outcomes in preclinical models [78]. HBM-Exos can also promote epithelial cell proliferation through p38 MAPK and cytoskeleton remodeling, significantly increasing the re-epithelialization rate of epithelial cells, which is crucial in the re-epithelialization stage of wound healing [79]. Their ability to modulate immune responses and repair tissue damage highlights their clinical potential in treating inflammatory wounds and supporting tissue regeneration.

#### 3.1.3 CM-Exos

CM-Exos are rich in antioxidants and exhibit potent anti-inflammatory effects [80]. CM-Exos have been shown to reduce oxidative stress and regulate inflammation, promoting faster tissue repair [81,82]. These exosomes modulate the immune response and facilitate wound healing, making them valuable in treating chronic wounds and mitigating inflammatory conditions.

#### 3.1.4 YM-Exos

Gao et al. found that YM-Exos could alleviate polysaccharide-induced intestinal inflammation by inhibiting PI3K/AKT/C3 pathway activation, and intestinal epithelial cells (IEC-6 cell line) treated with YM-Exos had a significantly higher cell survival rate under hypoxic conditions than treated BM-Exos [83]. The miRNAs of MDEs in the consumption cattle can regulate the proliferation of intestinal epithelial cells in the hypoxic environment. Although primarily studied for gastrointestinal health, their ability to improve cellular resilience under hypoxia suggests potential applications in wound healing for tissues exposed to reduced oxygen levels.

#### 3.1.5 PM-Exos

PM-Exos are notable for their potential in promoting intestinal cell proliferation and gut health in neonates [84]. PM-Exos contain high levels of non-coding RNAs, including miRNAs, that regulate digestive system development and immune function in piglets. PM-Exos increase intestinal cell proliferation, making them valuable for treating gastrointestinal diseases. Although studies on their direct impact on wound healing are limited, the regulatory role of PM-Exos in inflammatory pathways suggests potential applications in tissue regeneration [72].

#### 3.1.6 PAM-Exos

Although research on PAM-Exos is limited, early studies indicate they are enriched with immune-modulating miRNAs and antioxidants, which help reduce oxidative stress and promote tissue regeneration [85]. PAM-Exos may improve wound healing by modulating the immune response and protecting tissues from oxidative damage, although more quantitative studies are needed to fully understand their therapeutic potential.

#### 3.1.7 Rat MDEs (RM-Exos)

RM-Exos are emerging as a potential therapeutic agent for intestinal diseases. Studies show that RM-Exos significantly enhance intestinal epithelial cell viability, promoting stem cell activity and cell proliferation by inhibiting inflammatory pathways, specifically Toll-like receptor 4 (TLR4) activation [86]. While RM-Exos have demonstrated efficacy in gastrointestinal health, their therapeutic potential in wound healing is still under investigation.

### 3.2 MDE at different stages of lactation

**Fig. 4:**
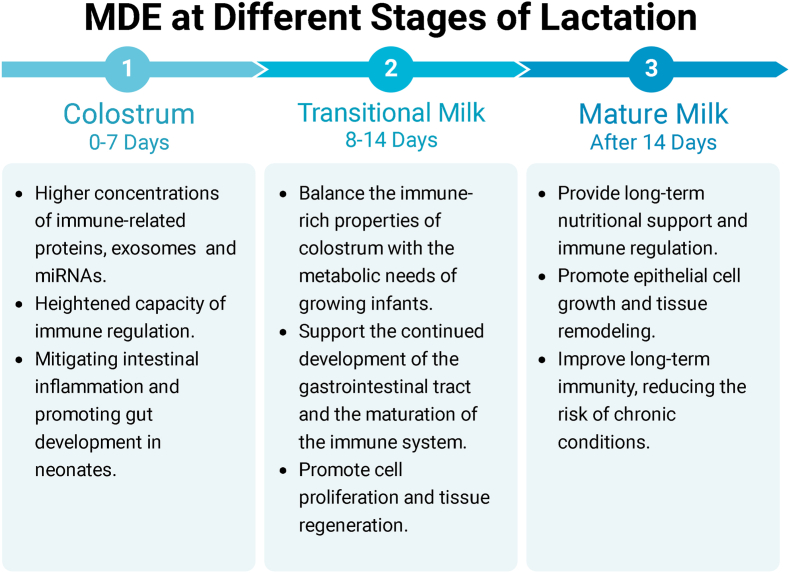
MDEs at different stages of lactation have different effects and have a profound impact on the therapeutic potential of wounds.

The composition and functionality of MDEs vary significantly across different stages of lactation. Key stages, colostrum, transitional milk, and mature milk, are characterized by distinct exosomal content, including variations in proteins, miRNAs, and immune-related molecules (Fig. 4). These differences have profound implications for the therapeutic potential of MDEs, particularly in immune modulation, tissue regeneration, and neonatal health.

#### 3.2.1 Col-MDEs

Colostrum, the first form of milk produced postpartum, is particularly rich in bioactive compounds and exosomes [53]. Studies show that Col-MDEs contain significantly higher concentrations of immune-related proteins and miRNAs than those from mature milk. For instance, bta-miR-221 in bovine Col-MDEs is 48-fold higher than in mature milk, indicating a heightened capacity for immune regulation during early lactation [87]. Col-MDEs have also been shown to reduce inflammatory markers, making them highly effective in mitigating intestinal inflammation and promoting gut development in neonates [88].

In humans, exosomes in colostrum are similarly enriched in immune-regulatory molecules. Studies have revealed that exosome concentrations are substantially higher in colostrum collected during 3–8 postnatal days (early lactation period) than in mature milk obtained during the second month postpartum [89,90]. Notably, miR-22-3p, a miRNA particularly abundant in human colostrum, has been identified as a key regulator in modulating neonatal immune responses and providing protection against infectious agents [87]. The enhanced immune-modulatory properties of Col-MDEs render them particularly valuable for supporting neonatal immune system development and for therapeutic applications in neonatal pathologies, including necrotizing enterocolitis [91].

#### 3.2.2 Transitional-MDEs

Transitional milk, produced after colostrum and before mature milk, contains exosomes that balance the immune-rich properties of colostrum with the metabolic needs of growing infants [92]. Quantitative data indicate that exosomes from transitional milk contain elevated levels of transport-related proteins, including Ras-related proteins (Rab-5B, Rab-5C, Rab-8A), which are essential for cellular signaling and growth regulation [93]. These exosomes support the continued development of the gastrointestinal tract and help in the maturation of the immune system [94].

Furthermore, transitional-MDEs exhibit a significant increase in growth factors compared to colostrum, making them crucial for cell proliferation and tissue regeneration [95]. The balance of immune modulation and growth promotion in transitional-MDEs makes them highly adaptive to the evolving needs of infants during this period.

#### 3.2.3 Mature-MDEs

As lactation progresses, mature-MDEs exhibit a different profile, focusing more on long-term nutritional support and immune regulation. In bovine mature milk, bta-miR-375 expression increases by 23-fold compared to colostrum, promoting intestinal epithelial proliferation and immune homeostasis [87].

In human mature milk, miR-141-3p is the most abundant, with its role centered around epithelial cell growth and tissue remodeling [87]. Mature-MDEs are also associated with long-term protection against infections, reducing the risk of chronic conditions like allergies and autoimmune diseases later in life [96].

The exosomal content of milk evolves significantly from colostrum to mature milk, with each stage playing a distinct role in immune regulation, cellular growth, and tissue repair. Col-MDEs are particularly rich in immune-modulating miRNAs, while transitional-MDEs and mature-MDEs support cell proliferation and long-term immune stability. The dynamic composition of MDEs at different lactation stages provides crucial insights into their potential therapeutic applications, particularly in neonatal health, immune therapy, and wound healing.

## 4 Isolation, extraction and sample pretreatment of MDEs

The successful isolation and extraction of MDEs are critical for maximizing their therapeutic and diagnostic potential. A variety of methods, including differential ulcentrifugation (DUC), density gradient ultracentrifugation (DG-UC), size-exclusion chromatography (SEC), ExoQuick™ kit, and microfluidic, have been utilized to isolate MDEs from complex biological matrices such as milk (Table 2). Each method offers distinct advantages based on parameters like exosome purity, recovery rate, sample handling, scalability, and impact on exosome composition and functionality [97]. Proper optimization and refinement of these methods are essential to preserve the structural integrity and functionality of exosomes, particularly for downstream applications in biomedicine.

**Table 2:** Methods for isolation of MDEs.

Isolation	Principle	Efficiency (Recovery Rate)	Advantages	Disadvantages	Ref
DUC	Isolation and purification of EVs based on sedimentation rate	Low (<20%)	1) Easy to use 2) Low cost	1) Requires large sample volume 2) Low recovery rate 3) Exosomes are prone to rupture and contamination 4) Time-consuming	[98]
DG-UC	Separation of exosomes based on size and density differences	Moderate (30–50%)	1) High recovery rate 2) High purity 3) Preserves exosome structure and function	1) Time consuming 2) Requires large sample volume 3) Potential for carrier damage and exosome aggregation	[99,100]
SEC	Separation of exosomes by size using a chromatography column	High (60–80%)	1) Protects biological functions of isolated exosomes from shear stress 2) High purity	1) Time-consuming 2) Not suitable for large-scale sample processing	[101,102]
ExoQuick kit	Isolation based on exosome density differences using polymer precipitation	Moderate (60–70%)	1) Simple operation 2) Suitable for small sample volume 3) Rapid processing 4) High recovery rate	1) Prone to protein contaminations 2) High cost	[98,103]
Microfluidic	Isolation based on immunoaffinity, size, or density using microfluidic chips	High (70–90%)	1) High purity 2) Rapid processing 3) Efficient for small sample volume	1) Requires sophisticated equipment 2) High cost	[[104], [105], [106], [107]]

### 4.1 DUC

DUC is widely used for exosomes isolation due to its simplicity and scalability. DUC exploits the sedimentation rate differences among cellular debris, large particles, and exosomes by subjecting milk samples to sequential centrifugation at increasing speeds [98]. Typically, raw milk undergoes a low-speed centrifugation step at 13,000 × *g* for 30 min to remove fat globules, and cell debris [108,109]. The supernatant is first centrifuged at 100,000 × *g* for 60 min at 4°C to remove larger particles and microbubbles. Subsequently, ultracentrifugation at 145,000 × *g* for 90 min at 4°C is performed to obtain a pellet of concentrated exosomes. The exosomes are then washed with phosphate-buffered saline (PBS) and filtered through a 0.22 μm membrane to ensure purity before stored at −80°C.

While DUC is an accessible and low-cost method, it suffers from low recovery rates (often below 20%) and can result in exosome damage due to high shear forces [19]. Co-precipitation of protein aggregates is another challenge, necessitating additional purification steps. These limitations can compromise exosome integrity, reducing their therapeutic efficacy. Despite these drawbacks, DUC remains the foundation for initial MDE isolation, particularly for large-scale studies. However, its low recovery rate and potential for exosome damage limit its suitability for clinical applications requiring high yields and intact exosome functionality.

### 4.2 DG-UC

DG-UC is the preferred technique for isolating MDEs due to its ability to achieve high-purity isolation based on density and size differentials [99]. The method utilizes a sucrose or iodixanol gradient, ensuring the separation of exosomes with minimal contamination, crucial for biomedical applications where exosome integrity is paramount.

In the DG-UC method, the milk sample is first centrifuged at 100,000 × *g* for 1 h to remove large particles. The resulting supernatant is layered onto a 10%–40% (w/v) sucrose or iodixanol gradient and centrifuged at 200,000 × *g* for 18 h. Exosomes typically accumulate in the 1.13–1.19 g/mL density range. After collection, exosomes are washed and concentrated to ensure high purity [110]. Exosomes isolated by DG-UC are generally between 50 and 100 nm in size, and the process ensures excellent structural preservation, making it the gold standard for isolating biologically active exosomes. Studies confirm that ultracentrifugation is the most widely used exosome separation method so far, accounting for 85% or even higher [100].

While DG-UC is highly effective, it has notable drawbacks, including extended processing time and the need for large sample volumes. Additionally, there is a risk of exosome aggregation if handling is not optimized. These limitations can affect the final yield and integrity of exosomes. Recent improvements include the combination of DG-UC with SEC, which has been shown to increase both yield and purity, ensuring better recovery without compromising exosome functionality and integrity [111].

DG-UC remains a cornerstone technique for isolating high-purity MDEs, balancing efficiency and precision. Its use in clinical and biomedical research is likely to expand as further optimizations enhance processing speed and scalability.

### 4.3 SEC

SEC is a highly efficient method for isolating MDEs based on their particle size. This technique is increasingly favored due to its ability to maintain exosome integrity and purity without subjecting samples to the high shear forces typical of ultracentrifugation. SEC is particularly valuable for separating exosomes from soluble proteins and other contaminants, making it ideal for both clinical and research applications.

The SEC process begins by first centrifuging milk at low speed to remove macroscopic particles and fat globules. The supernatant is then loaded onto a chromatography column, such as agarose CL-2B, for separation based on size. Exosomes are typically collected in fractions corresponding to their 50–150 nm size range. Studies have shown that SEC yields high-purity exosomes with low protein contamination, making it particularly effective for downstream proteomic analysis [101]. In practical applications, SEC can isolate up to 26 fractions per 0.5 mL of sample, with the peak fraction containing exosomes exhibiting a significantly lower protein concentration than other fractions. The remaining proteins are typically precipitated using trichloroacetic acid, and the purified exosomes are further validated through methods such as Western blotting, flow cytometry, and electron microscopy [44].

Recent innovations in SEC protocols have improved the yield and purity of exosomes. One such improvement includes combining SEC with polymer-based precipitation methods, which significantly enhances the ability to isolate distinct exosomal populations [112]. This approach allows for quantitative and qualitative analysis of exosomal protein and nucleic acid content, leading to better biomarker discovery and therapeutic applications. Studies comparing SEC to other methods, such as ultracentrifugation, have demonstrated that SEC not only offers better exosome recovery but also maintains functional vesicle integrity better, as it avoids the high centrifugal forces that can damage exosomes [102]. These characteristics make SEC a preferred method, particularly when the focus is on functional studies and clinical samples, where purity and exosome activity are critical.

### 4.4 ExoQuick™ kit

The ExoQuick™ kit, developed by System Biosciences (California, USA), provides a polymer-based precipitation method for isolating MDEs without the need for ultracentrifugation [98]. This approach is particularly advantageous for laboratories working with small sample volumes and those lacking access to high-speed ultracentrifugation equipment [94].

ExoQuick™ isolation begins by removing larger particles from the milk via centrifugation at 5000 × *g* for 30 min at 4°C. The resulting supernatant is then filtered through 10.0 μm, 0.45 μm, and 0.22 μm filters to eliminate further contaminants. ExoQuick™ reagent is mixed with the supernatant, followed by incubation for 12 h at 4°C. After incubation, centrifugation at 1,500 × *g* for 30 min results in exosome precipitation. The final pellet is resuspended in 100 μl PBS and can be analyzed using electron microscopy for exosome morphology and size validation [113].

The yield with ExoQuick™ has been shown to reach 60–70% of available exosomes in milk samples, making it a highly efficient method for preliminary studies or routine exosome collection. Additionally, it can enhance the recovery rate of exosomes, making it a preferred choice in many studies [97]. However, this method tends to co-precipitate protein contaminants, which can compromise the purity of the isolated exosomes and potentially affect their therapeutic efficacy [110]. For applications demanding higher purity, ExoQuick™ is often combined with SEC or DG-UC to improve the quality of the isolated exosomes. While ExoQuick™ provides a rapid and accessible method for exosome recovery, its scalability and suitability for clinical applications are limited by the need for additional purification steps.

### 4.5 Microfluidic

Microfluidic technology offers an advanced approach for isolating MDEs by using size-based separation and immunoaffinity techniques [114]. This method targets exosomal markers such as CD9, CD63, and CD81, which bind to antibodies immobilized on microchips, ensuring high specificity and purity [97].

Microfluidic devices are designed for handling small sample volumes with high precision, making them ideal for exosome isolation from milk. The platform's ability to process fluids at the microscale reduces sample loss and maintains exosome integrity [107]. With processing times as short as 30 min, microfluidics provides a faster alternative to traditional methods like ultracentrifugation while delivering similar or better recovery rates, often exceeding 70%, with purity levels reaching 90% or higher [105].

Despite their high efficiency, microfluidic devices are limited by complexity and cost, which have hindered widespread adoption [106]. These systems are still mostly used in research rather than routine clinical diagnostics due to the high expense of chip production and maintenance [115]. However, as the technology matures, it holds great promise for personalized medicine and biomarker discovery, particularly in the development of lab-on-a-chip systems capable of combining isolation, analysis, and diagnostic capabilities.

## 5 Application difference and optimization strategy of MDEs in different types of wounds

Wound healing can be broadly categorized into acute wounds (e.g., mechanical injuries, chemical wounds, surgical wounds) and chronic wounds (e.g., burns, infections, diabetic ulcers) [116]. The healing process involves distinct stages: hemostasis, inflammation, proliferation, and remodeling [7]. Each influenced by the wound's underlying pathology. Recent studies have highlighted the therapeutic potential of MDEs in wound management, offering a safe and scalable alternative to cell-derived exosomes [117].

MDEs exhibit unique advantages, including high biocompatibility, low immunogenicity, and superior stability in biological fluids [118]. These properties make MDEs particularly suitable for clinical applications in diverse wound types, promoting cell communication, reducing inflammation, and enhancing tissue regeneration [119]. Additionally, the high yield and stability of MDEs in harsh conditions, further underscore their potential as effective therapeutic agents [[120], [121], [122]]. Cytokines and signaling pathways are critical for tissue regeneration, not only allowing cells to function and communicate with each other, but also playing a leading role in immune responses, especially in the inflammatory stage, the recruitment and interaction of immune cells and the inflammatory factors secreted by them (such as interleukin (IL) and tumor necrosis factor (TNF)) are the key links of the immune response and almost determine the outcomes of regeneration [123]. At the molecular level, exosomes modulate inflammation by delivering anti-inflammatory miRNAs that regulate the NF-κB signaling pathway, such as miR-146a and miR-155, which can be transmitted between immune cells as promoters of inflammatory response and immunosuppressant mediators, respectively [124]. This reduces the production of pro-inflammatory cytokines such as IL-6 and IL-12 p40. In angiogenesis, exosomes deliver miR-126-3p, targeting SPRED-1 and PIK3R2, thereby activating the PI3K/AKT/mTOR signaling pathway, which promotes angiogenesis and inhibits cell apoptosis [125]. Also, MDEs regulate HIF-1α, a key transcription factor that upregulates pro-angiogenic gene expression under hypoxic conditions to promote angiogenesis [126,127]. Additionally, exosomes affect collagen synthesis by transmitting TGFβ, FGF-1, VEGF, etc., thereby regulating the expression of collagen (especially collagen 1 and collagen 3) and matrix metalloproteinases (MMPs), ensuring the balance of ECM deposition and remodeling [128,129].

Understanding the application differences of MDEs between acute and chronic wounds is critical for optimizing their therapeutic use. Tailored strategies for MDE delivery, dosage, and targeting mechanisms are essential to address the specific challenges posed by each wound type, maximizing their healing efficacy.

### 5.1 Acute wound

Acute wounds such as surgical incisions, burns, and traumatic injuries generally follow a predictable healing process characterized by rapid tissue repair and minimal complications. MDEs have shown significant efficacy in accelerating acute wound healing by regulating key pathways involved in inflammation, angiogenesis, and tissue remodeling, releasing clotting tissue factors (TF), promoting collagen synthesis, and ECM remodeling.

#### 5.1.1 TGFβ/Smad signaling pathway

The TGFβ/Smad signaling pathway is fundamental in regulating wound healing processes, particularly in epithelialization, collagen production, and ECM remodeling [130]. This pathway is mediated by Smad proteins, which act as intracellular effectors of TGFβ signals. The TGFβ family includes three key isoforms, TGFβ1, β2, and β3, each with distinct roles [131]. TGFβ1 and TGFβ2 are known to drive collagen synthesis and fibrosis, while TGFβ3 is crucial in promoting scar-free healing by limiting fibrotic tissue formation.

Smad proteins can be classified into three types: receptor-regulated Smads (R-Smads) like Smad2 and Smad3, common mediator Smad (Smad4), and inhibitory Smads (Smad6, Smad7) [132]. Upon activation, Smad3 typically promotes ECM formation and tissue repair, whereas Smad7 inhibits this process by acting as a negative regulator, reducing excessive collagen deposition [133]. This balance is essential for minimizing scarring and achieving effective wound repair.

In the context of MDEs, studies have shown that these vesicles can modulate the TGFβ/Smad pathway, promoting scar-free healing. For example, Ahn et al. demonstrated that MDEs exhibit anti-inflammatory properties by downregulating pro-inflammatory cytokines like IL-6 and TNF-α, while simultaneously influencing TGFβ signaling by activating latent TGFβ1 and upregulating TGFβ3 levels [133]. This shift enhances tissue repair while minimizing scar formation, making MDEs a promising tool in wound healing applications.

**Fig. 5:**
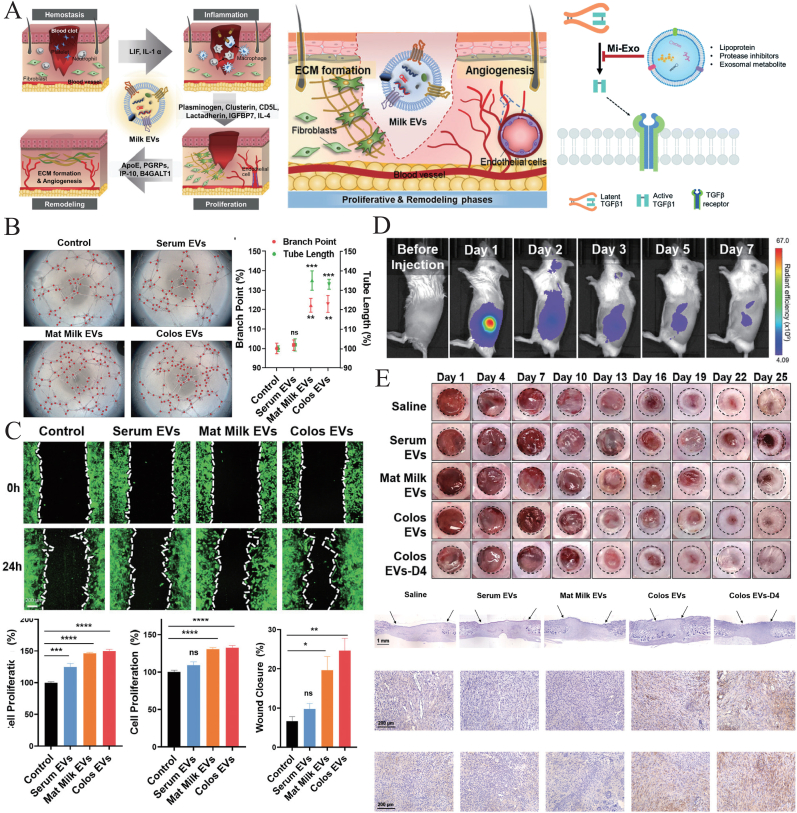
MDEs promote wound healing by regulating the TGFβ/Smad pathway. A) Schematic images of MDE-mediated wound healing and a possible model of TGF. Copyright 2021, Wiley-VCH. Copyright 2021, The Royal Society of Chemistry. B) Tube formation assays performed with MDEs treatment of SVEC4-10 cells (n = 5). Copyright 2021, Wiley-VCH. C) Wound scratch migration assay in NIH-3T3 cells. Copyright 2021, Wiley-VCH. D) Images of *in vivo* biodistribution analysis results obtained for 7 days after labeled Col-MDEs (n = 3). Copyright 2021, Wiley-VCH. E) Wound healing assay in different groups of mice and representative images of immunostaining for H&E, CD31 and elastin proteins in wound tissues from mice (n = 6). Copyright 2021, Wiley-VCH. All data are presented as mean ± SD (*∗p* < 0.05, *∗∗p* < 0.01, *∗∗∗p* < 0.001, *∗∗∗∗p* < 0.0001 vs control; Dunnett's multiple comparisons test).

Additionally, research by Kim et al. explored the therapeutic effects of MDEs from colostrum and mature milk in wound models (Fig. 5) [117]. They observed that MDEs reduced the expression of Smad7 and increased levels of TGFβ3, leading to enhanced ECM remodeling and improved healing outcomes. This suggests that MDE-mediated wound repair could be closely regulated through TGFβ/Smad modulation, making it a viable approach to accelerate healing and reduce scarring.

#### 5.1.2 Release of clotting TF

TF is a key regulator of the extrinsic coagulation pathway, typically absent from circulating blood but rapidly exposed upon vascular injury, triggering the formation of a fibrin clot to ensure hemostasis [134]. Under normal conditions, TF is primarily found in subendothelial tissues and plays a pivotal role in stopping bleeding following tissue trauma [135].

Interestingly, TF has also been identified in EVs from various biological fluids, including MDEs [136]. These vesicles carry TF on their surface, suggesting their active involvement in localized clot formation, even in non-blood compartments like saliva, amniotic fluid, and breast milk. Hu et al. found that MDEs exhibit significant pro-coagulant activity, aiding in the prevention of postpartum breast inflammation and supporting wound healing during breastfeeding by facilitating microvascular hemostasis [137].

This ability of MDEs to deliver TF at wound sites underscores their potential therapeutic role in wound management, especially for enhancing local clot formation and improving outcomes in both acute wounds and chronic conditions where hemostasis is compromised. The pro-coagulant properties of TF-exposing exosomes present a novel strategy for optimizing therapeutic interventions aimed at rapid clot formation and efficient tissue repair.

#### 5.1.3 Association with fibrous matrix

Nanofibrous materials mimic the nanoscale architecture of fibrin fibers in the ECM, providing a promising scaffold for hemostatic applications due to their high surface area, tunable porosity, and structural precision. Nanofibers, in particular, are known to facilitate platelet adhesion and activation, which enhances hemostasis and has led to their use in various wound healing studies [138,139].

In a study by Bui et al., MDEs-immobilized HA/Gel electrospun fibrous mats were designed to serve as an advanced wound therapy matrix [119]. This innovative system allowed the controlled release of MDEs, accelerating wound closure and improving tissue regeneration *in vivo*. The integration of MDEs with fibrous matrices not only stabilizes the exosomes for controlled release but also amplifies platelet activation, supporting hemostasis and enhancing acute wounds repair. This dual functionality highlights the potential of combining MDEs with nanofibrous scaffolds to create a synergistic therapeutic platform for effective wound management.

#### 5.1.4 Promote the repair of UV-damaged skin

UV radiation is a primary environmental factor causing skin damage by inducing oxidative stress, which affects the ECM and collagen integrity. This results in photoaging, decreased skin elasticity, and can even lead to carcinogenesis under prolonged exposure [140]. The excessive generation of ROS during UV exposure disrupts cellular components, accelerating skin aging through DNA mutations, protein degradation, and lipid peroxidation [141,142].

MDEs, especially those derived from bovine colostrum, have emerged as promising therapeutic agents for mitigating UV-induced skin damage through their multifaceted regenerative mechanisms. These exosomes demonstrate remarkable antioxidant capacity by significantly scavenging ROS, thereby protecting dermal fibroblasts from UV-induced oxidative stress and subsequent apoptosis. In addition, BM-Exos can induce type 1 collagen synthesis in human fibroblasts through the STAT 6 pathway, while donkey milk and human milk can promote cell cycle and proliferation of skin fibroblasts in vitro by activating growth regulatory kinases, especially p-ERK pathway [143,144]. Han et al. demonstrate that Col-MDEs not only play an antioxidant role by reducing intracellular ROS through the glutathione oxidation pathway, but also stimulate the synthesis of type I collagen and regulate the expression of MMPs. Promote balanced remodeling of ECM and restoration of skin structural integrity [76].

MDEs also enhance the skin's ability to recover by improving fibroblast survival under UV irradiation, a critical factor for maintaining skin elasticity and preventing premature aging [145]. These exosomes' ability to promote collagen synthesis and counteract ROS highlights their value as a natural therapeutic agent for treating UV-damaged skin and preventing long-term photoaging effects.

### 5.2 DM chronic wounds

Diabetic chronic wounds present significant therapeutic challenges primarily due to persistent inflammation, impaired angiogenesis, and delayed collagen deposition, which collectively contribute to delayed and often incomplete healing [146,147]. The prolonged inflammatory phase, coupled with endothelial dysfunction and microcirculatory issues, leads to compromised tissue regeneration and increased susceptibility to complications [[148], [149], [150]].

Compared to traditional wound management strategies, such as topical antibiotics, growth factor therapies, and surgical debridement, MDEs offer several distinct advantages. In the context of chronic wound management, MDEs can play a key role as part of an integrated care strategy. Recent advancements in regenerative medicine have identified MDEs as promising vehicles for delivering therapeutic molecules like miRNAs and siRNAs, which can solve these potential problems by delivery modulation of key pathways involved in wound healing. MDEs have demonstrated remarkable efficacy in promoting angiogenesis, modulating inflammation, and enhancing collagen synthesis, offering a novel approach to accelerating DM wound healing.

#### 5.2.1 miR-31-5p delivery

miR-31-5p is a key pro-angiogenic miRNA known for its ability to enhance endothelial cell proliferation, migration and angiogenesis [64]. This miRNA has significant therapeutic potential in the treatment of diabetic wounds, where impaired angiogenesis and vascular dysfunction are central to the delayed healing processes [[151], [152], [153]]. One of the critical targets of miR-31-5p is hypoxia-inducible factor 1α inhibitor (HIF1AN), a negative regulator of HIF-1α, which plays an essential role in promoting angiogenesis under hypoxic conditions typically observed in diabetic chronic wounds [126,127]. Targeting HIF1AN with miR-31-5p helps to upregulate HIF-1α activity, thereby restoring angiogenic responses and supporting wound healing [154,155].

**Fig. 6:**
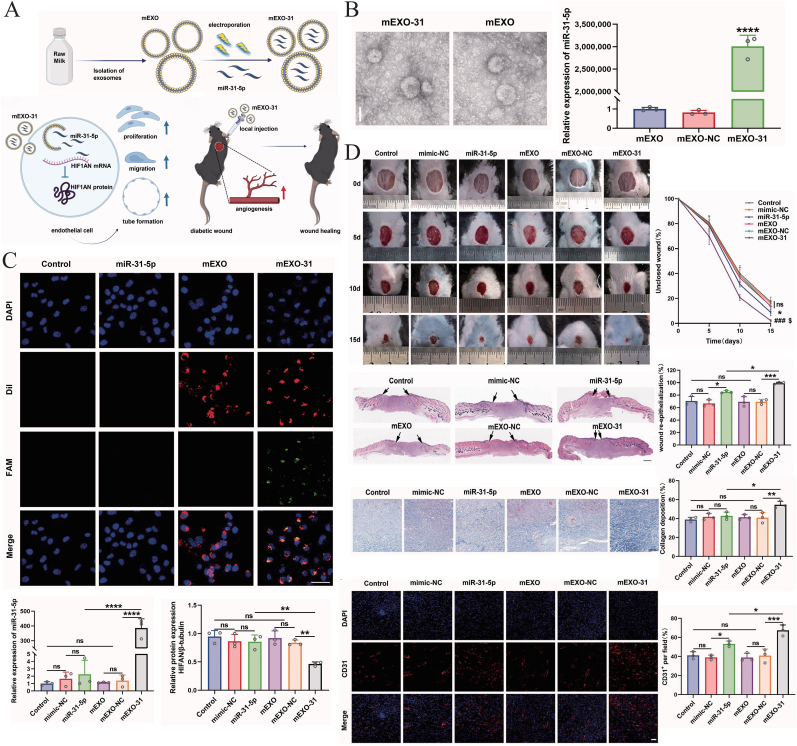
MDEs as a delivery system of miR-31-5p can accelerate the healing of diabetic wound. A) Schematic representation of MDEs loaded with miR-31-5p for wound treatment in mice. Copyright 2024, Taylor & Francis. B) TEM of MDEs and RT-PCR analysis of relative miR-31-5p levels. Scale bar, 50 μm. n = 3. Copyright 2024, Taylor & Francis. C) MDEs loaded with miR-31-5p could more effectively deliver miR-31-5p into cells. Scale bar, 50 μm. n = 3. Copyright 2024, Taylor & Francis. D) MDEs loaded with miR-31-5p can accelerate wound healing. Copyright 2024, Taylor & Francis. ns: no significant, *∗p* < 00.05, *∗∗p* < 00.01, *∗∗∗p <* 00.001, *∗∗∗∗p* < 00.0001. Data were presented as mean ± SD. One-way ANOVA with Tukey post-hoc test was used.

Recent studies by Yan et al. demonstrated that BM-Exos can serve as an effective delivery system for miR-31-5p (Fig. 6) [156]. Using electroporation, miR-31-5p mimics were encapsulated within BM-Exos, which not only improved cellular uptake but also protected miRNAs from degradation (Fig. 6A). In human umbilical vein endothelial cells (HUVECs), this exosomal delivery of miR-31-5p resulted in enhanced cell proliferation, migration, and tube formation, confirming its potent angiogenic effects (Fig. 6C).

*In vivo* experiments further validated the therapeutic efficacy of miR-31-5p-loaded exosomes, showing significant improvements in angiogenesis and accelerated healing in diabetic wound models (Fig. 6D) [157,158]. These findings highlight the potential of BM-Exos as a scalable, biocompatible delivery platform for miRNA-based therapies, specifically targeting the angiogenic deficiencies characteristic of diabetic wounds.

#### 5.2.2 siRNA-Keap1 delivery

Nuclear factor erythroid-2-related factor-2 (Nrf2) is a critical regulator of cellular responses to oxidative stress, which is a major contributor to impaired healing in diabetic wounds [159]. By controlling the expression of antioxidant enzymes, Nrf2 helps to reduce ROS and protect tissues from damage [160]. However, under diabetic conditions, Nrf2 activity is often suppressed due to the presence of Kelch-like ECH-associated protein 1 (Keap1), a repressor protein that sequesters Nrf2 in the cytoplasm [161]. Inhibiting Keap1 allows Nrf2 to activate its protective pathways, making it a promising target for therapeutic intervention.

The use of siRNA-Keap1 to knock down Keap1 expression and restore Nrf2 function offers a strategy to alleviate oxidative stress in diabetic wounds [162]. However, delivering siRNA effectively is challenging due to its instability and susceptibility to degradation. MDEs have emerged as an efficient and biocompatible vehicle for delivering siRNA, protecting it from degradation while facilitating cellular uptake. Xiang et al. demonstrated that siRNA-Keap1 could be effectively loaded into BM-Exos using sonication, resulting in a delivery system that significantly enhanced the therapeutic potential of siRNA [161]. The siKeap1-loaded exosomes improved endothelial cell proliferation and migration in vitro*,* along with a marked reduction in oxidative stress levels in HUVECs. In a preclinical diabetic wound model, this innovative delivery system accelerated wound healing, enhanced collagen formation, and promoted neovascularization, which are essential for proper tissue repair.

By targeting the Keap1-Nrf2 axis, MDE-mediated siRNA delivery provides a promising approach to restoring redox balance and promoting the resolution of chronic inflammation in diabetic wounds. This therapeutic paradigm underscores the potential of exosome-based therapies in advancing diabetic wound treatment, with the scalability, biocompatibility, and safety profile of MDEs positioning them as an exceptionally promising platform for siRNA-based interventions.

## 6 Challenges and strategies

**Fig. 7:**
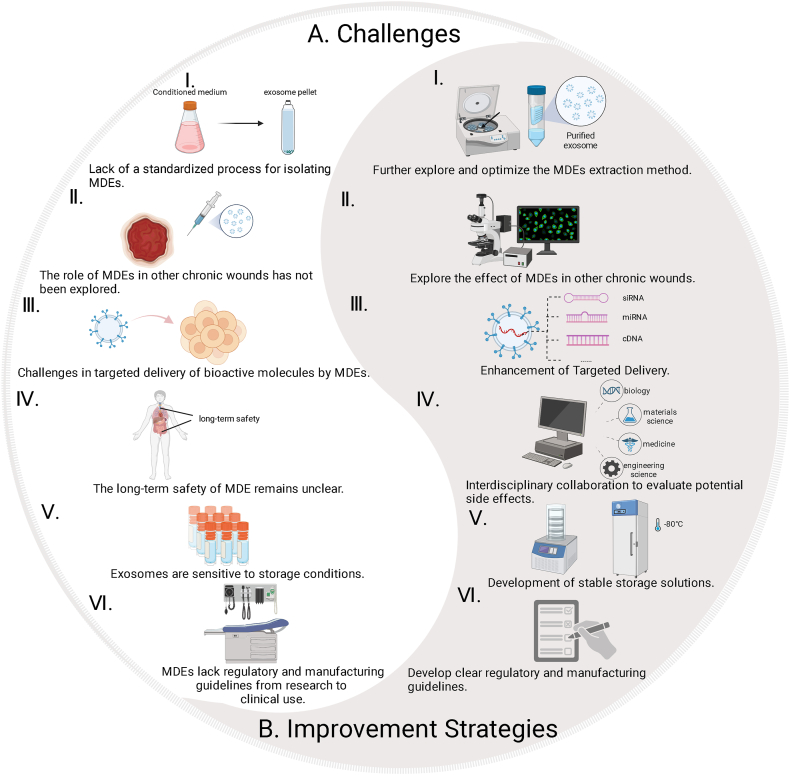
Current challenges of MDEs and corresponding strategies. The challenges (A) and corresponding improving strategies (B) of MDEs for wound healing.

Despite the remarkable therapeutic potential of MDEs in wound healing applications, as evidenced by their pro-angiogenic, anti-inflammatory, and tissue-regenerative properties, several critical challenges persist that impede their clinical translation and widespread therapeutic implementation. These challenges center around issues in isolation standardization, targeted delivery efficiency, long-term safety, storage stability and regulatory and manufacturing hurdles. Addressing these multifaceted challenges through approaches such as interdisciplinary research and technological innovation will be essential to optimize MDEs as reliable, safe, and effective therapeutic agents in wound management and regenerative medicine (Fig. 7).

(1) Inconsistent Isolation and Purification Methods: One of the most significant challenges is the lack of a standardized protocol for isolating MDEs [163]. Current methods, such as DUC and SEC, exhibit significant variability in terms of yield, purity, and functional integrity of the isolated exosomes [164]. This inconsistency not only affects the yield and biological functionality of the exosomes but also complicates comparative studies across laboratories. For MDEs to achieve clinical reliability, there is a need to develop uniform, reproducible isolation techniques that ensure the high purity and activity of MDEs without compromising their integrity [165]. In wound healing applications, achieving optimal exosome concentration and purity is essential, as these factors directly influence the therapeutic outcomes by modulating key cellular processes.
(2) Limited Application in Complex Wounds: While studies have shown that MDEs can facilitate healing in acute wounds and diabetic ulcers, their efficacy in chronic wound environments, such as pressure ulcers, venous ulcers, and infected wounds, remains underexplored. Chronic wounds present unique challenges, including persistent inflammation, microbial infection, and impaired angiogenesis, which may require more tailored exosomal interventions [166]. These challenges are further compounded by patient-specific factors such as age, gender, genetic background, existing health conditions like diabetes, and individual immune status, all of which can dramatically affect treatment outcomes [167,168]. For example, elderly patients or those with compromised immune systems might experience slower or less pronounced healing responses due to these chronic conditions [169]. This inter-individual variability necessitates a more customized approach to MDE therapy, ensuring that treatments are not only tailored to the general pathology of chronic wounds but also adapted to meet the specific needs of each patient [170]. Future research should focus on how MDEs can modulate these complex wound pathologies through anti-inflammatory and pro-angiogenic mechanisms and on understanding how individual differences impact long-term wound resolution.
(3) Targeted Delivery Challenges: The development of biomaterial therapies with precise and targeted immunomodulation is an important aspect to enhance tissue regeneration and immunomodulation [171]. Although MDEs offer superior biocompatibility and low immunogenicity, one of the key obstacles in their use for wound healing is achieving targeted delivery to the wound site [165]. Effective targeting ensures that bioactive molecules such as siRNAs, miRNAs, and growth factors are delivered precisely to the wound bed, enhancing therapeutic efficiency while minimizing systemic effects. Engineering MDEs for enhanced targeting—for instance, through surface modification or the incorporation of ligand-receptor systems—could increase their therapeutic effectiveness in complex wounds, where precise modulation of cell signaling pathways is crucial for regeneration [25,156,161].
(4) Safety Concerns and Long-Term Effects: Despite MDEs' demonstrated safety profile in short-term studies, their long-term safety in chronic wound applications remains uncertain. Concerns have been raised about the potential off-target effects of exosomal miRNAs, particularly those associated with conditions such as fibrosis, tumorigenesis, and inflammation. Specific miRNAs, like miR-21 and miR-155, have been implicated in tumor progression and immune dysregulation, thereby warranting careful consideration of their therapeutic application in chronic wound care [172]. Comprehensive long-term studies are necessary to assess whether repeated or high-dose applications of MDEs could lead to unwanted adverse effects in patients with chronic wounds.
(5) Storage Stability Challenges: Another critical barrier to the clinical adoption of MDEs is their storage stability. Exosomes are sensitive to environmental conditions such as temperature, pH, and freeze-thaw cycles, which can compromise their structural integrity and biological activity [173]. For instance, exposure to elevated temperatures (e.g., above 4°C) can lead to protein denaturation, resulting in reduced exosome functionality [174]. Current storage methods, including cryopreservation and addition of preservatives, have shown varying degrees of success in maintaining exosome functionality [175,176]. Cryopreservation at −80°C is commonly used but may lead to particle aggregation and reduced bioactivity over time. Lyophilization offers a promising alternative for long-term storage, but optimization of cryoprotectants (trehalose) and rehydration protocols is necessary to ensure consistent recovery of functional exosomes [177]. Addressing these storage challenges is essential for the development of stable, off-the-shelf MDE-based therapies.
(6) Regulatory and Manufacturing Challenges: The translation of MDEs from research to clinical use faces significant regulatory and manufacturing challenges. Current manufacturing practices for MDEs lack scalability and consistency, which are critical for large-scale production. Additionally, the absence of clear regulatory guidelines for exosome-based products complicates their approval process. These include concerns related to animal welfare, the sustainability of large-scale milk production, and the potential for cross-species transmission of pathogens [172,178]. Addressing these challenges will require collaboration between researchers, industry stakeholders, and regulatory bodies to establish standardized protocols and ensure compliance with regulatory requirements. The International Society for Extracellular Vesicles (ISEV) and the European Cooperation in Science and Technology (COST) have highlighted the safety, regulatory, manufacturing, and quality control processes that must be considered for exosome-based therapeutics, and have discussed strategies to promote their clinical application [178].

To address these challenges, the following strategies are recommended:

(1) Optimization of Exosome Isolation: Establishing standardized protocols for isolating MDEs with high purity and biological activity is critical [179]. Improved isolation methods will ensure consistent therapeutic outcomes in clinical settings.
(2) Expanded Research on Chronic Wounds: Exploring the effects of MDEs in chronic wound models beyond diabetic ulcers will provide insights into their broader application. This includes evaluating their role in modulating inflammation, promoting angiogenesis, and improving collagen deposition in chronic wound environments.
(3) Enhancement of Targeted Delivery: By using advanced genetic engineering techniques or in combination with molecular targeting strategies, MDEs can target wound sites more precisely [21]. Surface modifications, such as ligand functionalization or antibody conjugation, allow these vesicles to recognize and bind key receptors expressed in the wound microenvironment. In addition, molecular targeting approaches can be tailored to interact with specific cell types involved in wound healing, such as fibroblasts, keratinocytes, or immune cells, thereby promoting tissue regeneration and reducing inflammation.
(4) Comprehensive Safety Profiling: Rigorous multi-species and long-term studies must be conducted to assess the potential side effects of MDEs, especially related to miRNA-mediated off-target effects. Interdisciplinary collaboration between wound care specialists, molecular biologists, and toxicologists will be crucial for developing safe and effective MDE-based therapies.
(5) Development of Stable Storage Solutions: Research into advanced storage methods, such as optimized lyophilization protocols and novel cryoprotectants, is needed to improve the long-term stability of MDEs. Standardized storage conditions will facilitate the clinical translation of MDE-based therapies.
(6) Regulatory and Manufacturing Standardization: Developing scalable and reproducible manufacturing processes, normative ethical governance and clear regulatory guidelines, and exploring the potential of MDEs for wound treatment from a veterinary perspective such as their application in large-scale animal models and preclinical trials are essential for the clinical translation of MDEs. Collaboration between academia, industry, and regulatory agencies will be key to overcoming these hurdles.

## 7 Conclusion

MDEs have emerged as a promising therapeutic tool in the field of wound healing, offering unique advantages such as biocompatibility, low immunogenicity, and the capacity to deliver bioactive molecules essential for tissue repair. This review has highlighted the key roles of MDEs in both acute and diabetic chronic wound healing, illustrating their ability to modulate critical processes such as angiogenesis, collagen synthesis, and inflammatory regulation. In acute wounds, MDEs contribute to scar-free healing by influencing pathways like TGFβ/Smad, while in diabetic chronic wounds, they accelerate healing by delivering miRNAs and siRNAs that target impaired endothelial function and oxidative stress.

Despite their therapeutic potential, challenges remain in terms of standardizing isolation techniques, improving targeted delivery to wound sites, and ensuring the long-term safety of MDE applications. Future research should focus on optimizing these aspects to fully harness MDEs' potential in clinical settings. By addressing these challenges, MDEs could revolutionize the treatment of both acute and chronic wounds, providing a new generation of targeted therapies that improve healing outcomes and reduce complications.

## CRediT authorship contribution statement

**Jing Ruan:** Writing – original draft, Investigation. **Yuping Xia:** Writing – original draft. **Yilei Ma:** Writing – original draft. **Xiyao Xu:** Project administration. **Shihao Luo:** Resources. **Jia Yi:** Visualization. **Baihui Wu:** Resources. **Rongbing Chen:** Software. **Hanbing Wang:** Validation. **Honggang Yu:** Validation, Supervision. **Qinsi Yang:** Validation, Supervision. **Wei Wu:** Formal analysis, Conceptualization. **Da Sun:** Writing – review & editing, Funding acquisition, Conceptualization. **Junbo Zhong:** Writing – review & editing, Funding acquisition, Conceptualization.

## Declaration of competing interest

The authors declare that they have no known competing financial interests or personal relationships that could have appeared to influence the work reported in this paper.

## Data Availability

No data was used for the research described in the article.

## Abbreviation

(Alix): ALG-2 interacting protein X
(BM-Exos): Bovine milk exosomes
(Col-MDEs): Colostrum-derived exosomes
(CM-Exos): Camel MDEs
(circRNAs): Circular RNAs
(DG-UC): Density gradient ultracentrifugation
(DM): Diabetes mellitus
(DUC): Differential ulcentrifugation
(ECM): Extracellular matrix
(EVs): Extracellular vesicles
(HBM-Exos): Human breast milk-derived exosomes
(HUVECs): Human umbilical vein endothelial cells
(HIF1AN): Hypoxia-inducible factor 1α inhibitor
(IL): Interleukin
(Keap1): Kelch-like ECH-associated protein 1
(lncRNAs): Long non-coding RNAs
(miRNAs): MicroRNAs
(MDEs): Milk-derived exosomes
(Nrf2): Nuclear factor erythroid-2-related factor-2
(PAM-Exos): Panda MDEs
(PBS): Phosphate-buffered saline
(PC): Phosphatidylcholine
(PE): Phosphatidylethanolamine
(PS): Phosphatidylserine
(PM-Exos): Porcine MDEs
(RM-Exos): Rat MDEs
(ROS): Reactive oxygen species
(SEC): Size-exclusion chromatography
(SM): Sphingomyelin
(TF): Tissue factor
(TLR4): Toll-like receptor 4
(TNF): Tumor necrosis factor
(TSG101): Tumor susceptibility gene 101
(UV): Ultraviolet
(YM-Exos): Yak MDEs

## References

[1] Freedman B.R., Hwang C., Talbot S., Hibler B., Matoori S., Mooney D.J. (2023). Breakthrough treatments for accelerated wound healing. Sci. Adv..

[2] Ong K.L., Stafford L.K., McLaughlin S.A., Boyko E.J., Vollset S.E., Smith A.E., Dalton B.E., Duprey J., Cruz J.A., Hagins H., Lindstedt P.A., Aali A., Abate Y.H., Abate M.D., Abbasian M., Abbasi-Kangevari Z., Abbasi-Kangevari M., Abd ElHafeez S., Abd-Rabu R., Abdulah D.M., Abdullah A.Y.M., Abedi V., Abidi H., Aboagye R.G., Abolhassani H., Abu-Gharbieh E., Abu-Zaid A., Adane T.D., Adane D.E., Addo I.Y., Adegboye O.A., Adekanmbi V., Adepoju A.V., Adnani Q.E.S., Afolabi R.F., Agarwal G., Aghdam Z.B., Agudelo-Botero M., Aguilera Arriagada C.E., Agyemang-Duah W., Ahinkorah B.O., Ahmad D., Ahmad R., Ahmad S., Ahmad A., Ahmadi A., Ahmadi K., Ahmed A., Ahmed A., Ahmed L.A., Ahmed S.A., Ajami M., Akinyemi R.O., Al Hamad H., Al Hasan S.M., Al-Ahdal T.M.A., Alalwan T.A., Al-Aly Z., AlBataineh M.T., Alcalde-Rabanal J.E., Alemi S., Ali H., Alinia T., Aljunid S.M., Almustanyir S., Al-Raddadi R.M., Alvis-Guzman N., Amare F., Ameyaw E.K., Amiri S., Amusa G.A., Andrei C.L., Anjana R.M., Ansar A., Ansari G., Ansari-Moghaddam A., Anyasodor A.E., Arabloo J., Aravkin A.Y., Areda D., Arifin H., Arkew M., Armocida B., Ärnlöv J., Artamonov A.A., Arulappan J., Aruleba R.T., Arumugam A., Aryan Z., Asemu M.T., Asghari-Jafarabadi M., Askari E., Asmelash D., Astell-Burt T., Athar M., Athari S.S., Atout M.M.W., Avila-Burgos L., Awaisu A., Azadnajafabad S., B D.B., Babamohamadi H., Badar M., Badawi A., Badiye A.D., Baghcheghi N., Bagheri N., Bagherieh S., Bah S., Bahadory S., Bai R., Baig A.A., Baltatu O.C., Baradaran H.R., Barchitta M., Bardhan M., Barengo N.C., Bärnighausen T.W., Barone M.T.U., Barone-Adesi F., Barrow A., Bashiri H., Basiru A., Basu S., Basu S., Batiha A.-M.M., Batra K., Bayih M.T., Bayileyegn N.S., Behnoush A.H., Bekele A.B., Belete M.A., Belgaumi U.I., Belo L., Bennett D.A., Bensenor I.M., Berhe K., Berhie A.Y., Bhaskar S., Bhat A.N., Bhatti J.S., Bikbov B., Bilal F., Bintoro B.S., Bitaraf S., Bitra V.R., Bjegovic-Mikanovic V., Bodolica V., Boloor A., Brauer M., Brazo-Sayavera J., Brenner H., Butt Z.A., Calina D., Campos L.A., Campos-Nonato I.R., Cao Y., Cao C., Car J., Carvalho M., Castañeda-Orjuela C.A., Catalá-López F., Cerin E., Chadwick J., Chandrasekar E.K., Chanie G.S., Charan J., Chattu V.K., Chauhan K., Cheema H.A., Chekol Abebe E., Chen S., Cherbuin N., Chichagi F., Chidambaram S.B., Cho W.C.S., Choudhari S.G., Chowdhury R., Chowdhury E.K., Chu D.-T., Chukwu I.S., Chung S.-C., Coberly K., Columbus A., Contreras D., Cousin E., Criqui M.H., Cruz-Martins N., Cuschieri S., Dabo B., Dadras O., Dai X., Damasceno A.A.M., Dandona R., Dandona L., Das S., Dascalu A.M., Dash N.R., Dashti M., Dávila-Cervantes C.A., De la Cruz-Góngora V., Debele G.R., Delpasand K., Demisse F.W., Demissie G.D., Deng X., Denova-Gutiérrez E., Deo S.V., Dervišević E., Desai H.D., Desale A.T., Dessie A.M., Desta F., Dewan S.M.R., Dey S., Dhama K., Dhimal M., Diao N., Diaz D., Dinu M., Diress M., Djalalinia S., Doan L.P., Dongarwar D., dos Santos Figueiredo F.W., Duncan B.B., Dutta S., Dziedzic A.M., Edinur H.A., Ekholuenetale M., Ekundayo T.C., Elgendy I.Y., Elhadi M., El-Huneidi W., Elmeligy O.A.A., Elmonem M.A., Endeshaw D., Esayas H.L., Eshetu H.B., Etaee F., Fadhil I., Fagbamigbe A.F., Fahim A., Falahi S., Faris M.E.M., Farrokhpour H., Farzadfar F., Fatehizadeh A., Fazli G., Feng X., Ferede T.Y., Fischer F., Flood D., Forouhari A., Foroumadi R., Foroutan Koudehi M., Gaidhane A.M., Gaihre S., Gaipov A., Galali Y., Ganesan B., Garcia-Gordillo M., Gautam R.K., Gebrehiwot M., Gebrekidan K.G., Gebremeskel T.G., Getacher L., Ghadirian F., Ghamari S.-H., Ghasemi Nour M., Ghassemi F., Golechha M., Goleij P., Golinelli D., Gopalani S.V., Guadie H.A., Guan S.-Y., Gudayu T.W., Guimarães R.A., Guled R.A., Gupta R., Gupta K., Gupta V.B., Gupta V.K., Gyawali B., Haddadi R., Hadi N.R., Haile T.G., Hajibeygi R., Haj-Mirzaian A., Halwani R., Hamidi S., Hankey G.J., Hannan M.A., Haque S., Harandi H., Harlianto N.I., Hasan S.M.M., Hasan S.S., Hasani H., Hassanipour S., Hassen M.B., Haubold J., Hayat K., Heidari G., Heidari M., Hessami K., Hiraike Y., Holla R., Hossain S., Hossain M.S., Hosseini M.-S., Hosseinzadeh M., Hosseinzadeh H., Huang J., Huda M.N., Hussain S., Huynh H.-H., Hwang B.-F., Ibitoye S.E., Ikeda N., Ilic I.M., Ilic M.D., Inbaraj L.R., Iqbal A., Islam S.M.S., Islam R.M., Ismail N.E., Iso H., Isola G., Itumalla R., Iwagami M., Iwu C.C.D., Iyamu I.O., Iyasu A.N., Jacob L., Jafarzadeh A., Jahrami H., Jain R., Jaja C., Jamalpoor Z., Jamshidi E., Janakiraman B., Jayanna K., Jayapal S.K., Jayaram S., Jayawardena R., Jebai R., Jeong W., Jin Y., Jokar M., Jonas J.B., Joseph N., Joseph A., Joshua C.E., Joukar F., Jozwiak J.J., Kaambwa B., Kabir A., Kabthymer R.H., Kadashetti V., Kahe F., Kalhor R., Kandel H., Karanth S.D., Karaye I.M., Karkhah S., Katoto P.D., Kaur N., Kazemian S., Kebede S.A., Khader Y.S., Khajuria H., Khalaji A., Khan M.A., Khan M., Khan A., Khanal S., Khatatbeh M.M., Khater A.M., Khateri S., khorashadizadeh F., Khubchandani J., Kibret B.G., Kim M.S., Kimokoti R.W., Kisa A., Kivimäki M., Kolahi A.-A., Komaki S., Kompani F., Koohestani H.R., Korzh O., Kostev K., Kothari N., Koyanagi A., Krishan K., Krishnamoorthy Y., Kuate Defo B., Kuddus M., Kuddus M.A., Kumar R., Kumar H., Kundu S., Kurniasari M.D., Kuttikkattu A., La Vecchia C., Lallukka T., Larijani B., Larsson A.O., Latief K., Lawal B.K., Le T.T.T., Le T.T.B., Lee S.W.H., Lee M., Lee W.-C., Lee P.H., Lee S., Lee S.W., Legesse S.M., Lenzi J., Li Y., Li M.-C., Lim S.S., Lim L.-L., Liu X., Liu C., Lo C.-H., Lopes G., Lorkowski S., Lozano R., Lucchetti G., Maghazachi A.A., Mahasha P.W., Mahjoub S., Mahmoud M.A., Mahmoudi R., Mahmoudimanesh M., Mai A.T., Majeed A., Majma Sanaye P., Makris K.C., Malhotra K., Malik A.A., Malik I., Mallhi T.H., Malta D.C., Mamun A.A., Mansouri B., Marateb H.R., Mardi P., Martini S., Martorell M., Marzo R.R., Masoudi R., Masoudi S., Mathews E., Maugeri A., Mazzaglia G., Mekonnen T., Meshkat M., Mestrovic T., Miao Jonasson J., Miazgowski T., Michalek I.M., Minh L.H.N., Mini G., Miranda J.J., Mirfakhraie R., Mirrakhimov E.M., Mirza-Aghazadeh-Attari M., Misganaw A., Misgina K.H., Mishra M., Moazen B., Mohamed N.S., Mohammadi E., Mohammadi M., Mohammadian-Hafshejani A., Mohammadshahi M., Mohseni A., Mojiri-forushani H., Mokdad A.H., Momtazmanesh S., Monasta L., Moniruzzaman M., Mons U., Montazeri F., Moodi Ghalibaf A., Moradi Y., Moradi M., Moradi Sarabi M., Morovatdar N., Morrison S.D., Morze J., Mossialos E., Mostafavi E., Mueller U.O., Mulita F., Mulita A., Murillo-Zamora E., Musa K.I., Mwita J.C., Nagaraju S.P., Naghavi M., Nainu F., Nair T.S., Najmuldeen H.H.R., Nangia V., Nargus S., Naser A.Y., Nassereldine H., Natto Z.S., Nauman J., Nayak B.P., Ndejjo R., Negash H., Negoi R.I., Nguyen H.T.H., Nguyen D.H., Nguyen P.T., Nguyen V.T., Nguyen H.Q., Niazi R.K., Nigatu Y.T., Ningrum D.N.A., Nizam M.A., Nnyanzi L.A., Noreen M., Noubiap J.J., Nzoputam O.J., Nzoputam C.I., Oancea B., Odogwu N.M., Odukoya O.O., Ojha V.A., Okati-Aliabad H., Okekunle A.P., Okonji O.C., Okwute P.G., Olufadewa I.I., Onwujekwe O.E., Ordak M., Ortiz A., Osuagwu U.L., Oulhaj A., Owolabi M.O., Padron-Monedero A., Padubidri J.R., Palladino R., Panagiotakos D., Panda-Jonas S., Pandey A., Pandey A., Pandi-Perumal S.R., Pantea Stoian A.M., Pardhan S., Parekh T., Parekh U., Pasovic M., Patel J., Patel J.R., Paudel U., Pepito V.C.F., Pereira M., Perico N., Perna S., Petcu I.-R., Petermann-Rocha F.E., Podder V., Postma M.J., Pourali G., Pourtaheri N., Prates E.J.S., Qadir M.M.F., Qattea I., Raee P., Rafique I., Rahimi M., Rahimifard M., Rahimi-Movaghar V., Rahman M.O., Rahman M.A., Rahman M.H.U., Rahman M., Rahman M.M., Rahmani M., Rahmani S., Rahmanian V., Rahmawaty S., Rahnavard N., Rajbhandari B., Ram P., Ramazanu S., Rana J., Rancic N., Ranjha M.M.A.N., Rao C.R., Rapaka D., Rasali D.P., Rashedi S., Rashedi V., Rashid A.M., Rashidi M.-M., Ratan Z.A., Rawaf S., Rawal L., Redwan E.M.M., Remuzzi G., Rengasamy K.R., Renzaho A.M.N., Reyes L.F., Rezaei N., Rezaei N., Rezaeian M., Rezazadeh H., Riahi S.M., Rias Y.A., Riaz M., Ribeiro D., Rodrigues M., Rodriguez J.A.B., Roever L., Rohloff P., Roshandel G., Roustazadeh A., Rwegerera G.M., Saad A.M.A., Saber-Ayad M.M., Sabour S., Sabzmakan L., Saddik B., Sadeghi E., Saeed U., Saeedi Moghaddam S., Safi S., Safi S.Z., Saghazadeh A., Saheb Sharif-Askari N., Saheb Sharif-Askari F., Sahebkar A., Sahoo S.S., Sahoo H., Saif-Ur-Rahman K., Sajid M.R., Salahi S., Salahi S., Saleh M.A., Salehi M.A., Salomon J.A., Sanabria J., Sanjeev R.K., Sanmarchi F., Santric-Milicevic M.M., Sarasmita M.A., Sargazi S., Sathian B., Sathish T., Sawhney M., Schlaich M.P., Schmidt M.I., Schuermans A., Seidu A.-A., Senthil Kumar N., Sepanlou S.G., Sethi Y., Seylani A., Shabany M., Shafaghat T., Shafeghat M., Shafie M., Shah N.S., Shahid S., Shaikh M.A., Shanawaz M., Shannawaz M., Sharfaei S., Shashamo B.B., Shiri R., Shittu A., Shivakumar K.M., Shivalli S., Shobeiri P., Shokri F., Shuval K., Sibhat M.M., Silva L.M.L.R., Simpson C.R., Singh J.A., Singh P., Singh S., Siraj M.S., Skryabina A.A., Sohag A.A.M., Soleimani H., Solikhah S., Soltani-Zangbar M.S., Somayaji R., Sorensen R.J.D., Starodubova A.V., Sujata S., Suleman M., Sun J., Sundström J., Tabarés-Seisdedos R., Tabatabaei S.M., Tabatabaeizadeh S.-A., Tabish M., Taheri M., Taheri E., Taki E., Tamuzi J.J.L., Tan K.-K., Tat N.Y., Taye B.T., Temesgen W.A., Temsah M.-H., Tesler R., Thangaraju P., Thankappan K.R., Thapa R., Tharwat S., Thomas N., Ticoalu J.H.V., Tiyuri A., Tonelli M., Tovani-Palone M.R., Trico D., Trihandini I., Tripathy J.P., Tromans S.J., Tsegay G.M., Tualeka A.R., Tufa D.G., Tyrovolas S., Ullah S., Upadhyay E., Vahabi S.M., Vaithinathan A.G., Valizadeh R., van Daalen K.R., Vart P., Varthya S.B., Vasankari T.J., Vaziri S., verma Verma M., Verras G.-I., Vo D.C., Wagaye B., Waheed Y., Wang Z., Wang Y., Wang C., Wang F., Wassie G.T., Wei M.Y.W., Weldemariam A.H., Westerman R., Wickramasinghe N.D., Wu Y., Wulandari R.D., Xia J., Xiao H., Xu S., Xu X., Yada D.Y., Yang L., Yatsuya H., Yesiltepe M., Yi S., Yohannis H.K., Yonemoto N., You Y., Zaman S.B., Zamora N., Zare I., Zarea K., Zarrintan A., Zastrozhin M.S., Zeru N.G., Zhang Z.-J., Zhong C., Zhou J., Zielińska M., Zikarg Y.T., Zodpey S., Zoladl M., Zou Z., Zumla A., Zuniga Y.M.H., Magliano D.J., Murray C.J.L., Hay S.I., Vos T. (2023). Global, regional, and national burden of diabetes from 1990 to 2021, with projections of prevalence to 2050: a systematic analysis for the Global Burden of Disease Study 2021. Lancet.

[3] Gould J. (2018). Superpowered skin. Nature.

[4] Rodrigues M., Kosaric N., Bonham C.A., Gurtner G.C. (2019). Wound healing: a cellular perspective. Physiol. Rev..

[5] Hajhosseini B., Gurtner G.C., Sen C.K. (2019).

[6] Xiong Y., Mi B.-B., Shahbazi M.-A., Xia T., Xiao J. (2024). Microenvironment-responsive nanomedicines: a promising direction for tissue regeneration. Mil Med Res.

[7] Boulton A.J., Vileikyte L., Ragnarson-Tennvall G., Apelqvist J. (2005). The global burden of diabetic foot disease. Lancet.

[8] Howell-Jones R.S., Wilson M.J., Hill K.E., Howard A.J., Price P.E., Thomas D.W. (2005). A review of the microbiology, antibiotic usage and resistance in chronic skin wounds. J. Antimicrob. Chemother..

[9] Riedemann H.I., Schmidt M.F., Baron J.M. (2023). Therapy of pathological scars. J Dtsch Dermatol Ges.

[10] Wu W., Wang Y., Tang Z., Gao Y., Huo Y. (2020). Regulatory oversight of cell therapy in China: government's efforts in patient access and therapeutic innovation. Pharmacol. Res..

[11] You H.-J., Han S.-K. (2014). Cell therapy for wound healing. J. Kor. Med. Sci..

[12] El Andaloussi S., Mäger I., Breakefield X.O., Wood M.J.A. (2013). Extracellular vesicles: biology and emerging therapeutic opportunities. Nat. Rev. Drug Discov..

[13] Han G., Kim H., Jang H., Kim E.S., Kim S.H., Yang Y. (2024). Oral TNF-α siRNA delivery via milk-derived exosomes for effective treatment of inflammatory bowel disease. Bioact. Mater..

[14] Kim H.I., Park J., Zhu Y., Wang X., Han Y., Zhang D. (2024). Recent advances in extracellular vesicles for therapeutic cargo delivery. Exp. Mol. Med..

[15] Li Y., Zhu Z., Li S., Xie X., Qin L., Zhang Q., Yang Y., Wang T., Zhang Y. (2024). Exosomes: compositions, biogenesis, and mechanisms in diabetic wound healing. J. Nanobiotechnol..

[16] Kalluri R., LeBleu V.S. (2020). The biology, function, and biomedical applications of exosomes. Science.

[17] Golchin A., Farahany T.Z., Khojasteh A., Soleimanifar F., Ardeshirylajimi A. (2019). The clinical trials of mesenchymal stem cell therapy in skin diseases: an update and concise review. Curr. Stem Cell Res. Ther..

[18] Liang Y., Duan L., Lu J., Xia J. (2021). Engineering exosomes for targeted drug delivery. Theranostics.

[19] Desai N., Gadeval A., Kathar U., Sengupta P., Kalia K., Tekade R.K. (2021). Emerging roles and biopharmaceutical applications of milk derived exosomes. J. Drug Deliv. Sci. Technol..

[20] Li D., Yao X., Yue J., Fang Y., Cao G., Midgley A.C., Nishinari K., Yang Y. (2022). Advances in bioactivity of MicroRNAs of plant-derived exosome-like nanoparticles and milk-derived extracellular vesicles. J. Agric. Food Chem..

[21] Aqil F., Kausar H., Agrawal A.K., Jeyabalan J., Kyakulaga A.-H., Munagala R., Gupta R. (2016). Exosomal formulation enhances therapeutic response of celastrol against lung cancer. Exp. Mol. Pathol..

[22] Betker J.L., Angle B.M., Graner M.W., Anchordoquy T.J. (2019). The potential of exosomes from cow milk for oral delivery. J. Pharmaceut. Sci..

[23] Xu Q., Yang S., Zhang K., Liu Y., Li L., Qu S. (2024). Enhanced antibacterial activity of bovine milk exosome-based drug formulation against bacterial pathogens. Food Chem..

[24] Wu X., Shen J., Zhong Y., Zhao X., Zhou W., Gao P., Wang X., An W. (2024). Large-scale isolation of milk exosomes for skincare. Pharmaceutics.

[25] Munagala R., Aqil F., Jeyabalan J., Gupta R.C. (2016). Bovine milk-derived exosomes for drug delivery. Cancer Lett..

[26] Zempleni J., Sukreet S., Zhou F., Wu D., Mutai E. (2019). Milk-derived exosomes and metabolic regulation. Annu Rev Anim Biosci.

[27] Le Doare K., Holder B., Bassett A., Pannaraj P.S. (2018). Mother's milk: a purposeful contribution to the development of the infant microbiota and immunity. Front. Immunol..

[28] Sedykh S., Kuleshova A., Nevinsky G. (2020). Milk exosomes: perspective agents for anticancer drug delivery. Indian J. Manag. Sci..

[29] Skotland T., Hessvik N.P., Sandvig K., Llorente A. (2019). Exosomal lipid composition and the role of ether lipids and phosphoinositides in exosome biology. JLR (J. Lipid Res.).

[30] Record M., Carayon K., Poirot M., Silvente-Poirot S. (2014). Exosomes as new vesicular lipid transporters involved in cell–cell communication and various pathophysiologies. Biochim. Biophys. Acta Mol. Cell Biol. Lipids.

[31] Buratta S., Urbanelli L., Tognoloni A., Latella R., Cerrotti G., Emiliani C., Chiaradia E. (2023). Protein and lipid content of milk extracellular vesicles: a comparative overview. Life.

[32] Gurunathan S., Kang M.-H., Kim J.-H. (2021). A comprehensive review on factors influences biogenesis, functions, therapeutic and clinical implications of exosomes. Int. J. Nanomed..

[33] Donoso-Quezada J., Ayala-Mar S., González-Valdez J. (2021). The role of lipids in exosome biology and intercellular communication: function, analytics and applications. Traffic.

[34] Fu S., Wang Y., Xia X., Zheng J.C. (2020). Exosome engineering: current progress in cargo loading and targeted delivery. NanoImpact.

[35] Antimisiaris S.G., Mourtas S., Marazioti A. (2018). Exosomes and exosome-inspired vesicles for targeted drug delivery. Pharmaceutics.

[36] Tienda-Vázquez M.A., Hanel J.M., Márquez-Arteaga E.M., Salgado-Álvarez A.P., Scheckhuber C.Q., Alanis-Gómez J.R., Espinoza-Silva J.I., Ramos-Kuri M., Hernández-Rosas F., Melchor-Martínez E.M., Parra-Saldívar R. (2023). Exosomes: a promising strategy for repair, regeneration and treatment of skin disorders. Cells.

[37] Kong X., Patel N.A., Chalfant C.E., Cooper D.R. (2023). Ceramide synthesis regulates biogenesis and packaging of exosomal MALAT1 from adipose derived stem cells, increases dermal fibroblast migration and mitochondrial function. Cell Commun. Signal..

[38] Trajkovic K., Hsu C., Chiantia S., Rajendran L., Wenzel D., Wieland F., Schwille P., Brügger B., Simons M. (2008). Ceramide triggers budding of exosome vesicles into multivesicular endosomes. Science.

[39] Burgert A., Schlegel J., Bécam J., Doose S., Bieberich E., Schubert-Unkmeir A., Sauer M. (2017). Characterization of plasma membrane ceramides by super-resolution microscopy. Angew Chem. Int. Ed. Engl..

[40] Xie Q., Hao Y., Li N., Song H., Chen X., Zhou Z., Wang J., Zhang Y., Li H., Han P., Wang X. (2024). Cellular uptake of engineered extracellular vesicles: biomechanisms, engineered strategies, and disease treatment. Adv. Healthcare Mater..

[41] Pohl E.E., Jovanovic O. (2019). The role of phosphatidylethanolamine adducts in modification of the activity of membrane proteins under oxidative stress. Molecules.

[42] Yassin A.M., Abdel Hamid M.I., Farid O.A., Amer H., Warda M. (2016). Dromedary milk exosomes as mammary transcriptome nano-vehicle: their isolation, vesicular and phospholipidomic characterizations. J. Adv. Res..

[43] Skotland T., Sandvig K., Llorente A. (2017). Lipids in exosomes: current knowledge and the way forward. Prog. Lipid Res..

[44] Blans K., Hansen M.S., Sørensen L.V., Hvam M.L., Howard K.A., Möller A., Wiking L., Larsen L.B., Rasmussen J.T. (2017). Pellet-free isolation of human and bovine milk extracellular vesicles by size-exclusion chromatography. J. Extracell. Vesicles.

[45] Admyre C., Johansson S.M., Qazi K.R., Filén J.-J., Lahesmaa R., Norman M., Neve E.P.A., Scheynius A., Gabrielsson S. (2007). Exosomes with immune modulatory features are present in human breast milk. J. Immunol..

[46] Savina A., Fader C.M., Damiani M.T., Colombo M.I. (2005). Rab11 promotes docking and fusion of multivesicular bodies in a calcium-dependent manner. Traffic.

[47] Giovanazzi A., van Herwijnen M.J.C., Kleinjan M., van der Meulen G.N., Wauben M.H.M. (2023). Surface protein profiling of milk and serum extracellular vesicles unveils body fluid-specific signatures. Sci. Rep..

[48] Hurley J.H., Odorizzi G. (2012). Get on the exosome bus with ALIX. Nat. Cell Biol..

[49] Gross J.C., Chaudhary V., Bartscherer K., Boutros M. (2012). Active Wnt proteins are secreted on exosomes. Nat. Cell Biol..

[50] Luga V., Zhang L., Viloria-Petit A.M., Ogunjimi A.A., Inanlou M.R., Chiu E., Buchanan M., Hosein A.N., Basik M., Wrana J.L. (2012). Exosomes mediate stromal mobilization of autocrine Wnt-PCP signaling in breast cancer cell migration. Cell.

[51] Regimbeau M., Abrey J., Vautrot V., Causse S., Gobbo J., Garrido C. (2022). Heat shock proteins and exosomes in cancer theranostics. Semin. Cancer Biol..

[52] Rahman M.M., Takashima S., Kamatari Y.O., Badr Y., Kitamura Y., Shimizu K., Okada A., Inoshima Y. (2021). Proteomic profiling of milk small extracellular vesicles from bovine leukemia virus-infected cattle. Sci. Rep..

[53] Samuel M., Chisanga D., Liem M., Keerthikumar S., Anand S., Ang C.-S., Adda C.G., Versteegen E., Jois M., Mathivanan S. (2017). Bovine milk-derived exosomes from colostrum are enriched with proteins implicated in immune response and growth. Sci. Rep..

[54] Baietti M.F., Zhang Z., Mortier E., Melchior A., Degeest G., Geeraerts A., Ivarsson Y., Depoortere F., Coomans C., Vermeiren E., Zimmermann P., David G. (2012). Syndecan-syntenin-ALIX regulates the biogenesis of exosomes. Nat. Cell Biol..

[55] Zeng B., Chen T., Luo J., Xie M., Wei L., Xi Q., Sun J., Zhang Y. (2020). Exploration of long non-coding RNAs and circular RNAs in porcine milk exosomes. Front. Genet..

[56] Hata T., Murakami K., Nakatani H., Yamamoto Y., Matsuda T., Aoki N. (2010). Isolation of bovine milk-derived microvesicles carrying mRNAs and microRNAs. Biochem. Biophys. Res. Commun..

[57] Izumi H., Kosaka N., Shimizu T., Sekine K., Ochiya T., Takase M. (2012). Bovine milk contains microRNA and messenger RNA that are stable under degradative conditions. J. Dairy Sci..

[58] Del Pozo-Acebo L., Hazas M.-C.L. de L., Tomé-Carneiro J., Gil-Cabrerizo P., San-Cristobal R., Busto R., García-Ruiz A., Dávalos A. (2021). Bovine milk-derived exosomes as a drug delivery vehicle for miRNA-based therapy. Int. J. Mol. Sci..

[59] Chen X., Gao C., Li H., Huang L., Sun Q., Dong Y., Tian C., Gao S., Dong H., Guan D., Hu X., Zhao S., Li L., Zhu L., Yan Q., Zhang J., Zen K., Zhang C.-Y. (2010). Identification and characterization of microRNAs in raw milk during different periods of lactation, commercial fluid, and powdered milk products. Cell Res..

[60] Tili E., Michaille J.-J., Calin G.A. (2008). Expression and function of micro-RNAs in immune cells during normal or disease state. Int. J. Med. Sci..

[61] Izumi H., Tsuda M., Sato Y., Kosaka N., Ochiya T., Iwamoto H., Namba K., Takeda Y. (2015). Bovine milk exosomes contain microRNA and mRNA and are taken up by human macrophages. J. Dairy Sci..

[62] Torrez Lamberti M.F., Parker L.A., Gonzalez C.F., Lorca G.L. (2023). Pasteurization of human milk affects the miRNA cargo of EVs decreasing its immunomodulatory activity. Sci. Rep..

[63] Zempleni J., Mcguire M.K., O’connor D.l. (2021). Human Milk.

[64] Ma J., Yong L., Lei P., Li H., Fang Y., Wang L., Chen H., Zhou Q., Wu W., Jin L., Sun D., Zhang X. (2022). Advances in microRNA from adipose-derived mesenchymal stem cell-derived exosome: focusing on wound healing. J. Mater. Chem. B.

[65] Liu X., Jin S., Liu J., Xu X. (2023). MiR-223-3p overexpressed adipose mesenchymal stem cell-derived exosomes promote wound healing via targeting MAPK10. Acta Histochem..

[66] K H., LandénNing X. (2017). Non-coding RNAs: new players in skin wound healing. Adv. Wound Care.

[67] Zeng B., Chen T., Luo J.-Y., Zhang L., Xi Q.-Y., Jiang Q.-Y., Sun J.-J., Zhang Y.-L. (2021). Biological characteristics and roles of noncoding RNAs in milk-derived extracellular vesicles. Adv. Nutr..

[68] Gu Y., Li M., Wang T., Liang Y., Zhong Z., Wang X., Zhou Q., Chen L., Lang Q., He Z., Chen X., Gong J., Gao X., Li X., Lv X. (2012). Lactation-related microRNA expression profiles of porcine breast milk exosomes. PLoS One.

[69] Misir S., Wu N., Yang B.B. (2022). Specific expression and functions of circular RNAs. Cell Death Differ..

[70] Wang Y., Li D., Wang Y., Li M., Fang X., Chen H., Zhang C. (2019). The landscape of circular RNAs and mRNAs in bovine milk exosomes. J. Food Compos. Anal..

[71] Wang Y., Liu J., Ma J., Sun T., Zhou Q., Wang W., Wang G., Wu P., Wang H., Jiang L., Yuan W., Sun Z., Ming L. (2019). Exosomal circRNAs: biogenesis, effect and application in human diseases. Mol. Cancer.

[72] Chen T., Xi Q.-Y., Ye R.-S., Cheng X., Qi Q.-E., Wang S.-B., Shu G., Wang L.-N., Zhu X.-T., Jiang Q.-Y., Zhang Y.-L. (2014). Exploration of microRNAs in porcine milk exosomes. BMC Genom..

[73] Lin D., Chen T., Xie M., Li M., Zeng B., Sun R., Zhu Y., Ye D., Wu J., Sun J., Xi Q., Jiang Q., Zhang Y. (2020). Oral administration of bovine and porcine milk exosome alter miRNAs profiles in piglet serum. Sci. Rep..

[74] Ebaid H., Abdel-Salam B., Hassan I., Al-Tamimi J., Metwalli A., Alhazza I. (2015). Camel milk peptide improves wound healing in diabetic rats by orchestrating the redox status and immune response. Lipids Health Dis..

[75] Hemmati A.A., Larki-Harchegani A., Shabib S., Jalali A., Rezaei A., Housmand G. (2018). Wound healing property of milk in full thickness wound model of rabbit. Int. J. Surg..

[76] Han G., Kim H., Kim D.E., Ahn Y., Kim J., Jang Y.J., Kim K., Yang Y., Kim S.H. (2022). The potential of bovine colostrum-derived exosomes to repair aged and damaged skin cells. Pharmaceutics.

[77] Galley J.D., Besner G.E. (2020). The therapeutic potential of breast milk-derived extracellular vesicles. Nutrients.

[78] L T., E A., L J., Sa P., K C., P S., E C., Mr S. (2021). Non-coding RNAs in human breast milk: a systematic review. Front. Immunol..

[79] Zonneveld M.I., van Herwijnen M.J.C., Fernandez-Gutierrez M.M., Giovanazzi A., de Groot A.M., Kleinjan M., van Capel T.M.M., Sijts A.J.A.M., Taams L.S., Garssen J., de Jong E.C., Kleerebezem M., Nolte-’t Hoen E.N.M., Redegeld F.A., Wauben M.H.M. (2021). Human milk extracellular vesicles target nodes in interconnected signalling pathways that enhance oral epithelial barrier function and dampen immune responses. J. Extracell. Vesicles.

[80] Adriano B., Cotto N.M., Chauhan N., Jaggi M., Chauhan S.C., Yallapu M.M. (2021). Milk exosomes: nature's abundant nanoplatform for theranostic applications. Bioact. Mater..

[81] Badawy A.A., El-Magd M.A., AlSadrah S.A. (2018). Therapeutic effect of camel milk and its exosomes on MCF7 cells in vitro and in vivo. Integr. Cancer Ther..

[82] Abu-Farha M., Thanaraj T.A., Qaddoumi M.G., Hashem A., Abubaker J., Al-Mulla F. (2020). The role of lipid metabolism in COVID-19 virus infection and as a drug target. Int. J. Mol. Sci..

[83] Gao H.N., Guo H.Y., Zhang H., Xie X.L., Wen P.C., Ren F.Z. (2019). Yak-milk-derived exosomes promote proliferation of intestinal epithelial cells in an hypoxic environment. J. Dairy Sci..

[84] Zhang S., Chen F., Zhang Y., Lv Y., Heng J., Min T., Li L., Guan W. (2018). Recent progress of porcine milk components and mammary gland function. J. Anim. Sci. Biotechnol..

[85] Ma J., Wang C., Long K., Zhang H., Zhang J., Jin L., Tang Q., Jiang A., Wang X., Tian S., Chen L., He D., Li D., Huang S., Jiang Z., Li M. (2017). Exosomal microRNAs in giant panda (Ailuropoda melanoleuca) breast milk: potential maternal regulators for the development of newborn cubs. Sci. Rep..

[86] Hock A., Miyake H., Li B., Lee C., Ermini L., Koike Y., Chen Y., Määttänen P., Zani A., Pierro A. (2017). Breast milk-derived exosomes promote intestinal epithelial cell growth. J. Pediatr. Surg..

[87] Yun B., Kim Y., Park D.J., Oh S. (2021). Comparative analysis of dietary exosome-derived microRNAs from human, bovine and caprine colostrum and mature milk. J. Anim. Sci. Technol..

[88] Cui Z., Amevor F.K., Zhao X., Mou C., Pang J., Peng X., Liu A., Lan X., Liu L. (2023). Potential therapeutic effects of milk-derived exosomes on intestinal diseases. J. Nanobiotechnol..

[89] Torregrosa Paredes P., Gutzeit C., Johansson S., Admyre C., Stenius F., Alm J., Scheynius A., Gabrielsson S. (2014). Differences in exosome populations in human breast milk in relation to allergic sensitization and lifestyle. Allergy.

[90] Hatmal M.M., Al-Hatamleh M.A.I., Olaimat A.N., Alshaer W., Hasan H., Albakri K.A., Alkhafaji E., Issa N.N., Al-Holy M.A., Abderrahman S.M., Abdallah A.M., Mohamud R. (2022). Immunomodulatory properties of human breast milk: MicroRNA contents and potential epigenetic effects. Biomedicines.

[91] Gao R., Zhang R., Qian T., Peng X., He W., Zheng S., Cao Y., Pierro A., Shen C. (2019). A comparison of exosomes derived from different periods breast milk on protecting against intestinal organoid injury. Pediatr. Surg. Int..

[92] Crookenden M.A., Walker C.G., Peiris H., Koh Y., Almughlliq F., Vaswani K., Reed S., Heiser A., Loor J.J., Kay J.K., Meier S., Donkin S.S., Murray A., Dukkipati V.S.R., Roche J.R., Mitchell M.D. (2017). Effect of circulating exosomes from transition cows on Madin-Darby bovine kidney cell function. J. Dairy Sci..

[93] Yang M., Song D., Cao X., Wu R., Liu B., Ye W., Wu J., Yue X. (2017). Comparative proteomic analysis of milk-derived exosomes in human and bovine colostrum and mature milk samples by iTRAQ-coupled LC-MS/MS. Food Res. Int..

[94] Rashidi M., Bijari S., Khazaei A.H., Shojaei-Ghahrizjani F., Rezakhani L. (2022). The role of milk-derived exosomes in the treatment of diseases. Front. Genet..

[95] Qin W., Tsukasaki Y., Dasgupta S., Mukhopadhyay N., Ikebe M., Sauter E.R. (2016). Exosomes in human breast milk promote EMT. Clin. Cancer Res..

[96] Ma L., Huo Y., Tang Q., Wang X., Wang W., Wu D., Li Y., Chen L., Wang S., Zhu Y., Wang W., Liu Y., Xu N., Chen L., Yu G., Chen J. (2024). Human breast milk exosomal miRNAs are influenced by premature delivery and affect neurodevelopment. Mol. Nutr. Food Res..

[97] Sidhom K., Obi P.O., Saleem A. (2020). A review of exosomal isolation methods: is size exclusion chromatography the best option?. Int. J. Mol. Sci..

[98] Livshits M.A., Khomyakova E., Evtushenko E.G., Lazarev V.N., Kulemin N.A., Semina S.E., Generozov E.V., Govorun V.M. (2015). Isolation of exosomes by differential centrifugation: theoretical analysis of a commonly used protocol. Sci. Rep..

[99] Wijenayake S., Eisha S., Tawhidi Z., Pitino M.A., Steele M.A., Fleming A.S., McGowan P.O. (2021). Comparison of methods for pre-processing, exosome isolation, and RNA extraction in unpasteurized bovine and human milk. PLoS One.

[100] Xu W.-M., Li A., Chen J.-J., Sun E.-J. (2023). Research development on exosome separation technology. J. Membr. Biol..

[101] Monguió-Tortajada M., Gálvez-Montón C., Bayes-Genis A., Roura S., Borràs F.E. (2019). Extracellular vesicle isolation methods: rising impact of size-exclusion chromatography. Cell. Mol. Life Sci..

[102] Lane R.E., Korbie D., Trau M., Hill M.M. (2019). Optimizing size exclusion chromatography for extracellular vesicle enrichment and proteomic analysis from clinically relevant samples. Proteomics.

[103] Ding M., Wang C., Lu X., Zhang C., Zhou Z., Chen X., Zhang C.-Y., Zen K., Zhang C. (2018). Comparison of commercial exosome isolation kits for circulating exosomal microRNA profiling. Anal. Bioanal. Chem..

[104] Yang D., Zhang W., Zhang H., Zhang F., Chen L., Ma L., Larcher L.M., Chen S., Liu N., Zhao Q., Tran P.H.L., Chen C., Veedu R.N., Wang T. (2020). Progress, opportunity, and perspective on exosome isolation - efforts for efficient exosome-based theranostics. Theranostics.

[105] Raju D., Bathini S., Badilescu S., Ghosh A., Packirisamy M. (2022). Microfluidic platforms for the isolation and detection of exosomes: a brief review. Micromachines.

[106] Chen C., Skog J., Hsu C.-H., Lessard R.T., Balaj L., Wurdinger T., Carter B.S., Breakefield X.O., Toner M., Irimia D. (2010). Microfluidic isolation and transcriptome analysis of serum microvesicles. Lab Chip.

[107] Wu Y., Wang Y., Lu Y., Luo X., Huang Y., Xie T., Pilarsky C., Dang Y., Zhang J. (2022). Microfluidic technology for the isolation and analysis of exosomes. Micromachines.

[108] Aqil F., Munagala R., Jeyabalan J., Agrawal A.K., Kyakulaga A.-H., Wilcher S.A., Gupta R.C. (2019). Milk exosomes - natural nanoparticles for siRNA delivery. Cancer Lett..

[109] Tao H., Xu H., Zuo L., Li C., Qiao G., Guo M., Zheng L., Leitgeb M., Lin X. (2020). Exosomes-coated bcl-2 siRNA inhibits the growth of digestive system tumors both in vitro and in vivo. Int. J. Biol. Macromol..

[110] Yamada T., Inoshima Y., Matsuda T., Ishiguro N. (2012). Comparison of methods for isolating exosomes from bovine milk. J. Vet. Med. Sci..

[111] Vaswani K., Koh Y.Q., Almughlliq F.B., Peiris H.N., Mitchell M.D. (2017). A method for the isolation and enrichment of purified bovine milk exosomes. Reprod. Biol..

[112] Martínez-Greene J.A., Hernández-Ortega K., Quiroz-Baez R., Resendis-Antonio O., Pichardo-Casas I., Sinclair D.A., Budnik B., Hidalgo-Miranda A., Uribe-Querol E., Ramos-Godínez M.D.P., Martínez-Martínez E. (2021). Quantitative proteomic analysis of extracellular vesicle subgroups isolated by an optimized method combining polymer-based precipitation and size exclusion chromatography. J. Extracell. Vesicles.

[113] Maburutse B.E., Park M.-R., Oh S., Kim Y. (2017). Evaluation and characterization of milk-derived microvescicle isolated from bovine colostrum. Food Science of Animal Resources.

[114] Lu M., Xing H., Yang Z., Sun Y., Yang T., Zhao X., Cai C., Wang D., Ding P. (2017). Recent advances on extracellular vesicles in therapeutic delivery: challenges, solutions, and opportunities. Eur. J. Pharm. Biopharm..

[115] Ashcroft B.A., de Sonneville J., Yuana Y., Osanto S., Bertina R., Kuil M.E., Oosterkamp T.H. (2012). Determination of the size distribution of blood microparticles directly in plasma using atomic force microscopy and microfluidics. Biomed. Microdevices.

[116] Qi L., Zhang C., Wang B., Yin J., Yan S. (2022). Progress in hydrogels for skin wound repair. Macromol. Biosci..

[117] Kim H., Kim D.E., Han G., Lim N.R., Kim E.H., Jang Y., Cho H., Jang H., Kim K.H., Kim S.H., Yang Y. (2022). Harnessing the natural healing power of colostrum: bovine milk-derived extracellular vesicles from colostrum facilitating the transition from inflammation to tissue regeneration for accelerating cutaneous wound healing. Adv. Healthcare Mater..

[118] Somiya M., Yoshioka Y., Ochiya T. (2018). Biocompatibility of highly purified bovine milk-derived extracellular vesicles. J. Extracell. Vesicles.

[119] Bui H.-T.D., You G., Lee M., Mao W., So C., Byeon C., Hong S., Mok H., Yoo H.S. (2024). Milk exosome-infused fibrous matrix for treatment of acute wound. J. Contr. Release.

[120] Liao Y., Du X., Li J., Lönnerdal B. (2017). Human milk exosomes and their microRNAs survive digestion in vitro and are taken up by human intestinal cells. Mol. Nutr. Food Res..

[121] Baddela V.S., Nayan V., Rani P., Onteru S.K., Singh D. (2016). Physicochemical biomolecular insights into Buffalo milk-derived nanovesicles. Appl. Biochem. Biotechnol..

[122] Carobolante G., Mantaj J., Ferrari E., Vllasaliu D. (2020). Cow milk and intestinal epithelial cell-derived extracellular vesicles as systems for enhancing oral drug delivery. Pharmaceutics.

[123] Li S., Lu L., Xiong Y., Xiao J. (2025). Nanomedicine-based immunotherapy for tissue regeneration, Burns & Trauma. tkaf015.

[124] Alexander M., Hu R., Runtsch M.C., Kagele D.A., Mosbruger T.L., Tolmachova T., Seabra M.C., Round J.L., Ward D.M., O'Connell R.M. (2015). Exosome-delivered microRNAs modulate the inflammatory response to endotoxin. Nat. Commun..

[125] Qu Q., Liu L., Cui Y., Liu H., Yi J., Bing W., Liu C., Jiang D., Bi Y. (2022). miR-126-3p containing exosomes derived from human umbilical cord mesenchymal stem cells promote angiogenesis and attenuate ovarian granulosa cell apoptosis in a preclinical rat model of premature ovarian failure. Stem Cell Res. Ther..

[126] Rey S., Semenza G.L. (2010). Hypoxia-inducible factor-1-dependent mechanisms of vascularization and vascular remodelling. Cardiovasc. Res..

[127] Hong W.X., Hu M.S., Esquivel M., Liang G.Y., Rennert R.C., McArdle A., Paik K.J., Duscher D., Gurtner G.C., Lorenz H.P., Longaker M.T. (2014). The role of hypoxia-inducible factor in wound healing. Adv. Wound Care.

[128] Noh C.H., Park S., Seong H.-R., Lee A., Tsolmon K.-E., Geum D., Hong S.-C., Kim T.M., Choi E.-K., Kim Y.-B. (2023). An exosome-rich conditioned medium from human amniotic membrane stem cells facilitates wound healing via increased reepithelization, collagen synthesis, and angiogenesis. Cells.

[129] White L.A., Mitchell T.I., Brinckerhoff C.E. (2000). Transforming growth factor β inhibitory element in the rabbit matrix metalloproteinase-1 (collagenase-1) gene functions as a repressor of constitutive transcription. Biochim. Biophys. Acta Gene Struct. Expr..

[130] Han A., Bandyopadhyay B., Jayaprakash P., Lua I., Sahu D., Chen M., Woodley D.T., Li W. (2012). The anti-motility signaling mechanism of TGFβ3 that controls cell traffic during skin wound healing. Biology Open.

[131] Lichtman M.K., Otero-Vinas M., Falanga V. (2016). Transforming growth factor beta (TGF-β) isoforms in wound healing and fibrosis. Wound Repair Regen..

[132] Derynck R., Zhang Y.E. (2003). Smad-dependent and Smad-independent pathways in TGF-beta family signalling. Nature.

[133] Ahn G., Kim Y.-H., Ahn J.-Y. (2021). Multifaceted effects of milk-exosomes (Mi-Exo) as a modulator of scar-free wound healing. Nanoscale Adv..

[134] van den Besselaar A.M.H.P., Chantarangkul V., Angeloni F., Binder N.B., Byrne M., Dauer R., Gudmundsdottir B.R., Jespersen J., Kitchen S., Legnani C., Lindahl T.L., Manning R.A., Martinuzzo M., Panes O., Pengo V., Riddell A., Subramanian S., Szederjesi A., Tantanate C., Herbel P., Tripodi A. (2018). International collaborative study for the calibration of proposed International Standards for thromboplastin, rabbit, plain, and for thromboplastin, recombinant, human, plain. J. Thromb. Haemostasis.

[135] Butenas S., Bouchard B.A., Brummel-Ziedins K.E., Parhami-Seren B., Mann K.G. (2005). Tissue factor activity in whole blood. Blood.

[136] Grover S.P., Mackman N. (2018). Tissue factor: an essential mediator of hemostasis and trigger of thrombosis. Arterioscler. Thromb. Vasc. Biol..

[137] Hu Y., Hell L., Kendlbacher R.A., Hajji N., Hau C., van Dam A., Berckmans R.J., Wisgrill L., Ay C., Pabinger I., Brisson A., Repa A., Nieuwland R., Thaler J. (2020). Human milk triggers coagulation via tissue factor-exposing extracellular vesicles. Blood Adv.

[138] Lu X., Li X., Yu J., Ding B. (2022). Nanofibrous hemostatic materials: structural design, fabrication methods, and hemostatic mechanisms. Acta Biomater..

[139] Wan L.-S., Xu Z.-K. (2009). Polymer surfaces structured with random or aligned electrospun nanofibers to promote the adhesion of blood platelets. J. Biomed. Mater. Res..

[140] Mohania D., Chandel S., Kumar P., Verma V., Digvijay K., Tripathi D., Choudhury K., Mitten S.K., Shah D. (2017). Ultraviolet radiations: skin defense-damage mechanism. Adv. Exp. Med. Biol..

[141] Hakozaki T., Date A., Yoshii T., Toyokuni S., Yasui H., Sakurai H. (2008). Visualization and characterization of UVB-induced reactive oxygen species in a human skin equivalent model. Arch. Dermatol. Res..

[142] Halliday G.M. (2005). Inflammation, gene mutation and photoimmunosuppression in response to UVR-induced oxidative damage contributes to photocarcinogenesis. Mutat. Res..

[143] Kippenberger S., Zöller N., Kleemann J., Müller J., Kaufmann R., Hofmann M., Bernd A., Meissner M., Valesky E. (2015). STAT6-Dependent collagen synthesis in human fibroblasts is induced by bovine milk. PLoS One.

[144] Kocic H., Langerholc T., Kostic M., Stojanovic S., Najman S., Krstic M., Nesic I., Godic A., Wollina U. (2020). The regenerative potential of donkey and human milk on the redox-sensitive and proliferative signaling pathways of skin fibroblasts. Oxid. Med. Cell. Longev..

[145] Wu J.-Y., Wu S.-N., Zhang L.-P., Zhao X.-S., Li Y., Yang Q.-Y., Yuan R.-Y., Liu J.-L., Mao H.-J., Zhu N.-W. (2022). Stem cell-derived exosomes: a new method for reversing skin aging. Tissue. Eng. Reg. Med..

[146] Chang M., Nguyen T.T. (2021). Strategy for treatment of infected diabetic foot ulcers. Acc. Chem. Res..

[147] Grennan D. (2019). Diabetic foot ulcers. JAMA.

[148] Davis F.M., Kimball A., Boniakowski A., Gallagher K. (2018). Dysfunctional wound healing in diabetic foot ulcers: new crossroads. Curr. Diabetes Rep..

[149] Zubair M., Ahmad J. (2019). Role of growth factors and cytokines in diabetic foot ulcer healing: a detailed review. Rev. Endocr. Metab. Disord..

[150] Ma J., Fang Y., Yu H., Yi J., Ma Y., Lei P., Yang Q., Jin L., Wu W., Li H., Sun D. (2024). Recent advances in living algae seeding wound dressing: focusing on diabetic chronic wound healing. Adv. Funct. Mater..

[151] Wu Y.-H., Hu T.-F., Chen Y.-C., Tsai Y.-N., Tsai Y.-H., Cheng C.-C., Wang H.-W. (2011). The manipulation of miRNA-gene regulatory networks by KSHV induces endothelial cell motility. Blood.

[152] Wang H.-W., Huang T.-S., Lo H.-H., Huang P.-H., Lin C.-C., Chang S.-J., Liao K.-H., Tsai C.-H., Chan C.-H., Tsai C.-F., Cheng Y.-C., Chiu Y.-L., Tsai T.-N., Cheng C.-C., Cheng S.-M. (2014). Deficiency of the microRNA-31-microRNA-720 pathway in the plasma and endothelial progenitor cells from patients with coronary artery disease. Arterioscler. Thromb. Vasc. Biol..

[153] Wong H.-K.A., Fatimy R.E., Onodera C., Wei Z., Yi M., Mohan A., Gowrisankaran S., Karmali P., Marcusson E., Wakimoto H., Stephens R., Uhlmann E.J., Song J.S., Tannous B., Krichevsky A.M. (2015). The cancer genome atlas analysis predicts MicroRNA for targeting cancer growth and vascularization in glioblastoma. Mol. Ther..

[154] Lando D., Peet D.J., Gorman J.J., Whelan D.A., Whitelaw M.L., Bruick R.K. (2002). FIH-1 is an asparaginyl hydroxylase enzyme that regulates the transcriptional activity of hypoxia-inducible factor. Genes Dev..

[155] Mahon P.C., Hirota K., Semenza G.L. (2001). FIH-1: a novel protein that interacts with HIF-1alpha and VHL to mediate repression of HIF-1 transcriptional activity. Genes Dev..

[156] Yan C., Chen J., Wang C., Yuan M., Kang Y., Wu Z., Li W., Zhang G., Machens H.-G., Rinkevich Y., Chen Z., Yang X., Xu X. (2022). Milk exosomes-mediated miR-31-5p delivery accelerates diabetic wound healing through promoting angiogenesis. Drug Deliv..

[157] Huang M., Nguyen P., Jia F., Hu S., Gong Y., de Almeida P.E., Wang L., Nag D., Kay M.A., Giaccia A.J., Robbins R.C., Wu J.C. (2011). Double knockdown of prolyl hydroxylase and factor-inhibiting hypoxia-inducible factor with nonviral minicircle gene therapy enhances stem cell mobilization and angiogenesis after myocardial infarction. Circulation.

[158] Umezu T., Tadokoro H., Azuma K., Yoshizawa S., Ohyashiki K., Ohyashiki J.H. (2014). Exosomal miR-135b shed from hypoxic multiple myeloma cells enhances angiogenesis by targeting factor-inhibiting HIF-1. Blood.

[159] Wu S., Liao X., Zhu Z., Huang R., Chen M., Huang A., Zhang J., Wu Q., Wang J., Ding Y. (2022). Antioxidant and anti-inflammation effects of dietary phytochemicals: the Nrf2/NF-κB signalling pathway and upstream factors of Nrf2. Phytochemistry.

[160] Iorio R., Celenza G., Petricca S. (2022). Multi-target effects of ß-caryophyllene and carnosic acid at the crossroads of mitochondrial dysfunction and neurodegeneration: from oxidative stress to microglia-mediated neuroinflammation. Antioxidants.

[161] Xiang X., Chen J., Jiang T., Yan C., Kang Y., Zhang M., Xiang K., Guo J., Jiang G., Wang C., XiangXu null, Yang X., Chen Z. (2023). Milk-derived exosomes carrying siRNA-KEAP1 promote diabetic wound healing by improving oxidative stress. Drug Deliv Transl Res.

[162] Liu Q., Zhang Y., Huang J., Xu Z., Li X., Yang J., Huang H., Tang S., Chai Y., Lin J., Yang C., Liu J., Lin S. (2022). Mesoporous silica-coated silver nanoparticles as ciprofloxacin/siRNA carriers for accelerated infected wound healing. J. Nanobiotechnol..

[163] Li P., Kaslan M., Lee S.H., Yao J., Gao Z. (2017). Progress in exosome isolation techniques. Theranostics.

[164] Lai J.J., Chau Z.L., Chen S.-Y., Hill J.J., Korpany K.V., Liang N.-W., Lin L.-H., Lin Y.-H., Liu J.K., Liu Y.-C., Lunde R., Shen W.-T. (2022). Exosome processing and characterization approaches for research and technology development. Adv. Sci..

[165] Kimiz-Gebologlu I., Oncel S.S. (2022). Exosomes: large-scale production, isolation, drug loading efficiency, and biodistribution and uptake. J. Contr. Release.

[166] Hade M.D., Suire C.N., Mossell J., Suo Z. (2022). Extracellular vesicles: emerging frontiers in wound healing. Med. Res. Rev..

[167] Gawronska-Kozak B., Kopcewicz M., Machcinska-Zielinska S., Walendzik K., Wisniewska J., Drukała J., Wasniewski T., Rutkowska J., Malinowski P., Pulinski M. (2023). Gender differences in post-operative human skin. Biomedicines.

[168] Beyene R.T., Derryberry S.L., Barbul A. (2020). The effect of comorbidities on wound healing. Surg. Clin..

[169] Kremer M., Burkemper N. (2024). Aging skin and wound healing. Clin. Geriatr. Med..

[170] Yang G.H., Lee Y.B., Kang D., Choi E., Nam Y., Lee K.H., You H.-J., Kang H.J., An S.H., Jeon H. (2021). Overcome the barriers of the skin: exosome therapy. Biomater. Res..

[171] Xiong Y., Mi B.-B., Lin Z., Hu Y.-Q., Yu L., Zha K.-K., Panayi A.C., Yu T., Chen L., Liu Z.-P., Patel A., Feng Q., Zhou S.-H., Liu G.-H. (2022). The role of the immune microenvironment in bone, cartilage, and soft tissue regeneration: from mechanism to therapeutic opportunity. Mil Med Res.

[172] Melnik B.C., Schmitz G. (2019). Exosomes of pasteurized milk: potential pathogens of Western diseases. J. Transl. Med..

[173] Yuan F., Li Y.-M., Wang Z. (2021). Preserving extracellular vesicles for biomedical applications: consideration of storage stability before and after isolation. Drug Deliv..

[174] Lee M., Ban J.-J., Im W., Kim M. (2016). Influence of storage condition on exosome recovery. Biotechnol Bioproc E.

[175] Wang H., Wu D., Sukreet S., Delaney A., Belfort M.B., Zempleni J. (2022). Quantitation of exosomes and their MicroRNA cargos in frozen human milk. JPGN Rep.

[176] Leiferman A., Shu J., Upadhyaya B., Cui J., Zempleni J. (2019). Storage of extracellular vesicles in human milk, and MicroRNA profiles in human milk exosomes and infant formulas. J. Pediatr. Gastroenterol. Nutr..

[177] Charoenviriyakul C., Takahashi Y., Nishikawa M., Takakura Y. (2018). Preservation of exosomes at room temperature using lyophilization. Int. J. Pharm..

[178] Lener T., Gimona M., Aigner L., Börger V., Buzas E., Camussi G., Chaput N., Chatterjee D., Court F.A., del Portillo H.A., O'Driscoll L., Fais S., Falcon-Perez J.M., Felderhoff-Mueser U., Fraile L., Gho Y.S., Görgens A., Gupta R.C., Hendrix A., Hermann D.M., Hill A.F., Hochberg F., Horn P.A., de Kleijn D., Kordelas L., Kramer B.W., Krämer-Albers E.-M., Laner-Plamberger S., Laitinen S., Leonardi T., Lorenowicz M.J., Lim S.K., Lötvall J., Maguire C.A., Marcilla A., Nazarenko I., Ochiya T., Patel T., Pedersen S., Pocsfalvi G., Pluchino S., Quesenberry P., Reischl I.G., Rivera F.J., Sanzenbacher R., Schallmoser K., Slaper-Cortenbach I., Strunk D., Tonn T., Vader P., van Balkom B.W.M., Wauben M., Andaloussi S.E., Théry C., Rohde E., Giebel B. (2015). Applying extracellular vesicles based therapeutics in clinical trials – an ISEV position paper. J. Extracell. Vesicles.

[179] Liu W.-Z., Ma Z.-J., Kang X.-W. (2022). Current status and outlook of advances in exosome isolation. Anal. Bioanal. Chem..

